# Revealing the Physiological Patterns of Dinoflagellates in North‐Eastern Adriatic Phytoplankton

**DOI:** 10.1002/ece3.72835

**Published:** 2026-01-04

**Authors:** Mia Knjaz, Ana Baricevic, Mirta Smodlaka Tankovic, Natasa Kuzat, Ivan Vlasicek, Lana Grizancic, Ivan Podolsak, Tjasa Kogovsek, Ariana Turkovic, Martin Pfannkuchen, Daniela Maric Pfannkuchen

**Affiliations:** ^1^ Center for Marine Research Ruder Boskovic Institute Rovinj Croatia

**Keywords:** community secession, marine ecosystem, metabolic activity, metatranscriptomics, phytoplankton

## Abstract

The northern Adriatic is a highly dynamic marine ecosystem where multiple environmental stressors, particularly phosphorus limitation, shape phytoplankton communities. Previous studies have established annual phytoplankton succession patterns primarily using light microscopy, while metatranscriptomic analyses have been lacking. This study used a metatranscriptomic approach to investigate the taxonomic and functional dynamics of the northern Adriatic phytoplankton community, focusing on the predominant group of dinoflagellates. Monthly sampling from April 2021 to March 2022 at two coastal stations revealed dinoflagellates as the most metabolically active phylum throughout the year in the size fraction > 50 μm. Peaks in metabolic activity of other studied phyla aligned with the characteristic seasonal species succession observed in previous studies. Community ordination indicated distinct seasonal shifts driven by environmental factors, notably phosphorus and silicon availability, as well as species interactions. Both photosynthesis and phagotrophy emerged as important trophic strategies for dinoflagellates. Nitrogen and phosphorus metabolism showed clear seasonal trends, with dinoflagellates employing various strategies for nutrient acquisition and recycling depending on resource availability. Changes in the activation of different cellular processes highlighted a seasonal shift in metabolic investment, with spring favouring rapid population expansion, while the rest of the year was characterised by prevalent transcription of genes indicative of cellular maintenance and adaptation. This study provides critical new insights into dinoflagellate phytoplankton ecology and emphasises the need for further multi‐method research to fully understand their role in the northern Adriatic ecosystem.

## Graphical abstract

Using metatranscriptomics, we have described the annual taxonomic and functional succession of the phytoplankton community in the north‐eastern Adriatic Sea, taking into account the effects of environmental factors on succession. The results obtained in this way represent the first comprehensive physiological characterisation of the phytoplankton community in the area studied. They are of great importance for answering fundamental questions of the phytoplankton ecology of the north‐eastern Adriatic Sea and at the same time enable the continuation of research on a broader spatial and temporal scale.

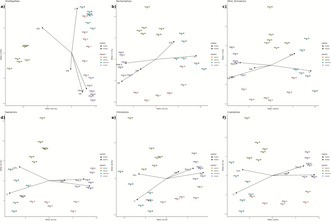

## 1 Introduction

Microbial eukaryotes (protists) play a central role in marine ecosystem functioning, as their metabolic activity supports numerous ecosystem functions and services. Phytoplankton, a fundamental component of eukaryotic microbial communities, utilises autotrophy (photosynthesis) and mixotrophy as trophic strategies (Flynn et al. 2019; Stoecker et al. 2017). These communities form the basis of marine food webs, and their biological activity contributes significantly to biogeochemical nutrient cycling and oxygen production (Carradec et al. 2018; De Vargas et al. 2015). Characterising the marine microbial community in terms of the taxonomic affiliation and functional role of individual members is an extremely complex and challenging task in marine ecology research. Members of the marine microbial community display a wide diversity of morphological shapes and sizes and possess various physiological mechanisms that reflect their trophic strategies, responses to changing environmental conditions and interactions both within and between communities (Caron et al. 2012).

Phosphorus is an important limiting factor for phytoplankton growth and development in many marine ecosystems (Dyhrman and Ruttenberg 2006; Lin et al. 2016). Consequently, research has focused on investigating the physiological responses and adaptation mechanisms of phytoplankton to varying phosphorus availability. A wide range of physiological responses has been identified that enable phytoplankton to cope with phosphorus limitation. These include changes in the activation of membrane transporters for inorganic phosphorus (orthophosphate, ${\mathrm{PO}}_{4}^{3-}$) and the acquisition of organic phosphorus compounds (dissolved organic phosphorus, DOP) from the environment through the activity of alkaline phosphatase enzymes as primary physiological responses to fluctuating phosphorus availability (Dyhrman and Ruttenberg 2006; Ivančić et al. 2021). Other adaptive strategies include polyphosphate storage, lipid accumulation, intracellular nutrient recycling and a shift towards phagotrophic nutrition (Jeong et al. 2010; Martin et al. 2011). These responses are recognised as important adaptations to phosphorus limitation. However, most of this research has been conducted using traditional methods such as light microscopic identification of species and spectroscopic detection of enzyme reactions, while omics studies (genomics and transcriptomics) have generally been scarce for marine microbial communities.

Transcriptomics enables the simultaneous characterisation of thousands of transcripts from a species or community (metatranscriptomics), providing detailed insight into the activity of the species or community and the life strategies present in the studied environment (Cohen et al. 2022; Lowe et al. 2017) by direct analysis of the species or community RNA. In recent years, metatranscriptomics has been successfully applied to studies of marine phytoplankton community physiological responses to various environmental conditions and the characterisation of temporal functional succession (Alexander, Jenkins, et al. 2015; Alexander, Rouco, et al. 2015; Cohen et al. 2017, 2021; Lampe et al. 2018, 2019; Zhang et al. 2019). Still, metatranscriptomics has limitations due to the complexity of microbial communities (high diversity and relative ratios of members), the large dynamic range of transcript expression, the short half‐life of RNA and several technology‐specific factors (Moran et al. 2013; Shakya et al. 2019; Wilms 2021).

Previous research has shown that the availability of dissolved phosphate (P) is one of the main factors influencing the community composition, abundance and expressed physiological patterns of phytoplankton in the northern Adriatic (Grilli et al. 2020; Ivančić et al. 2021). The seasonal hydrodynamics of the northern Adriatic, combined with inputs from the Po River, strongly influence the availability of nutrients essential for phytoplankton growth (Degobbis and Gilmartin 1990; Ivančić et al. 2012). The physiological response of the northern Adriatic phytoplankton to varying phosphorus conditions, considered the main factor determining phytoplankton growth and development, has been studied through in situ measurements of alkaline phosphatase activity (Ivančić et al. 2010, 2012, 2016, 2021), physiological in vitro (Kužat et al. 2022; Tanković et al. 2018; Vrana et al. 2023) and mesocosm (Malfatti et al. 2014) experiments.

Nutrient (phosphorus) limitation, a characteristic stressor for phytoplankton in the northern Adriatic, can also induce mucilage production by phytoplankton cells (Urbani et al. 2005). The massive formation of macroaggregates, referred to as the ‘mucilage phenomenon’, is an ecological disturbance known in the northern Adriatic. At the end of the 20th and the first decade of the 21st century, mucilage outbreaks were frequent in the northern Adriatic (Degobbis et al. 1999; Najdek et al. 2002). Mucilage macroaggregates are a complex mixture of organic compounds, with phytoplankton cells being the main source of organic precursors (Pistocchi et al. 2005; Totti et al. 2005). This organic matter is produced and accumulates under conditions of sharply rising temperatures, nutrient availability and relatively calm oceanographic conditions.

The succession of phytoplankton in the northern Adriatic has been studied using light microscopy methods for many years. A characteristic seasonal succession has been identified; however, under changing environmental conditions, deviations from these characteristic patterns in species occurrence and abundance can occur and have been reported to be more frequent and significant in the last decade (Aubry et al. 2004, 2012; Cerino et al. 2019; Godrijan et al. 2013; Marić et al. 2012; Mozetič et al. 2012; Totti et al. 2019; Vlašiček et al. 2025). The most abundant and extensively studied phytoplankton groups include diatoms (Bacillariophyta), dinoflagellates (Dinoflagellata) and haptophytes (Haptophyta). Long‐term succession studies suggest that diatoms dominate the community structure for most of the year (Aubry et al. 2004, 2012; Godrijan et al. 2013; Marić et al. 2012). Seasonal diatom blooms have been well‐documented, with species of the genus *Skeletonema* regularly blooming in winter and early spring, coinciding with rising nutrient concentrations (Aubry et al. 2004; Marić Pfannkuchen et al. 2018). An autumn diatom bloom has also been observed consistently (Godrijan et al. 2013; Marić et al. 2012; Neri et al. 2022). The contribution of dinoflagellates to the northern Adriatic phytoplankton community has generally been low, with notable increases in abundance occurring in June–July, following the spring diatom bloom when the water becomes nutrient‐depleted (Aubry et al. 2004). The seasonal succession of haptophytes in the northern Adriatic is characterised by two abundance peaks: the primary peak occurs in winter (December–February), corresponding to winter mixing, while a secondary peak occurs in May–June, coinciding with increasing light intensity and the onset of seasonal stratification (Cerino et al. 2017). Other phytoplankton groups, mostly consisting of smaller flagellates (‘phytoflagellates’), have rarely been the primary focus of phytoplankton studies in the area. Still, several studies have highlighted their importance in an overall phytoplankton abundance (Aubry et al. 2012; Cerino et al. 2017; Marić et al. 2012; Neri et al. 2022). Only recently, molecular studies, based on metabarcoding (sequencing of whole community molecular marker gene), have been used to investigate composition and succession of marine microbial eukaryotes in the northern Adriatic (Armeli Minicante et al. 2019; Cordier et al. 2019; Grižančić et al. 2023; Neri et al. 2025). These studies have confirmed the long‐standing paradigm of diatom‐dinoflagellate prevalence in the northern Adriatic while also shedding new light on the importance of the dinoflagellate community fraction. Still, for an accurate metabarcoding quantification of phytoplankton, along with methodological biases (Van Der Loos and Nijland 2021), the presence of 18S SSU rRNA gene copy number variation within species, genera and groups should be considered, including dinoflagellates and diatoms (Martin et al. 2022).

The aim of this study was to describe the taxonomic and functional succession of the northern Adriatic phytoplankton community (size fraction > 50 μm) using metatranscriptomics. This study includes data from two sampling stations where phytoplankton succession has been monitored for many years using light microscopy, enabling comparison of the newly collected metatranscriptomics data with previous community characterisation of the area. Additionally, by correlating the measured environmental parameters with the recorded temporal expression patterns, our study helps to determine the environmental factors affecting phytoplankton functional and community succession.

## 2 Materials and Methods

### 2.1 Study Area

The northern Adriatic is a shallow marine region of the Adriatic Sea, situated at the northernmost edge of the Mediterranean basin. This semi‐enclosed, shallow sea, with its unique hydrogeographic characteristics, forms a highly dynamic marine ecosystem. Along the eastern coast of the northern Adriatic, the Eastern Adriatic Current (EAC) transports high‐salinity, oligotrophic water into the northern Adriatic basin, while from the western coast, the Po River delivers low‐salinity, nutrient‐rich water into the basin (Degobbis et al. 2000). The Po River is one of the largest net contributors of freshwater to the entire Mediterranean (Degobbis and Gilmartin 1990). Additionally, it is characterised by a significantly higher concentration of nitrogen (N) compared with phosphorus (P) (Cozzi and Giani 2011), often resulting in an imbalanced N:P ratio in the northern Adriatic basin.

### 2.2 Sampling and RNA Isolation

**FIGURE 1:**
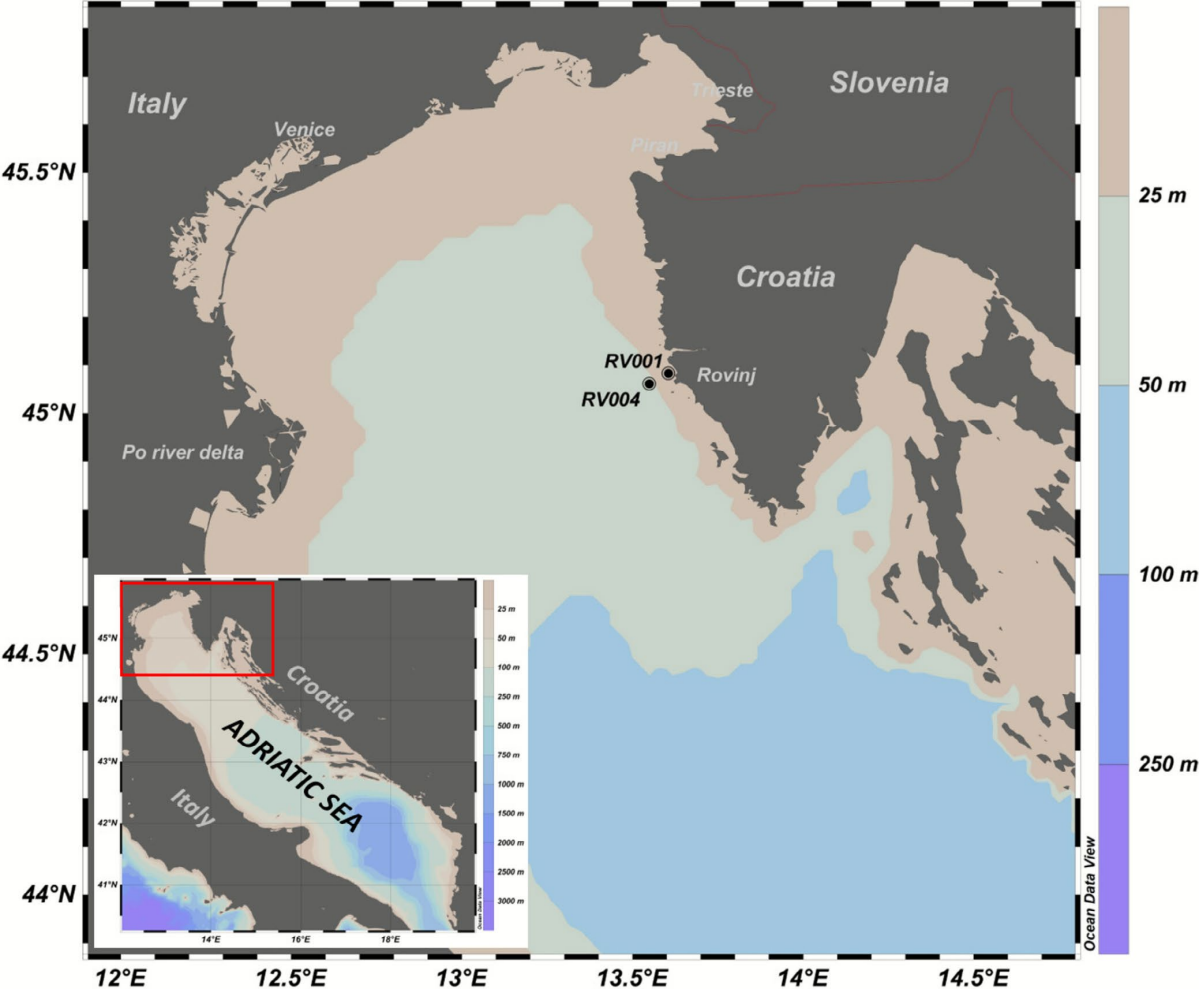
Sampling area. Stations RV001, RV004 where environmental data and biological samples for metatranscriptomics were collected.

Monthly sampling cruises were conducted from April 2021 to March 2022 at two research stations situated 1 NM (RV001, 45°04′48.0″N 13°36′36.0″E) and 3 NM (RV004, 45°04′35.2″N 13°34′05.1″E) from the coast (Figure 1). Both stations are part of the long‐term monitoring programme of the northern Adriatic, situated at a depth of approximately 30 m, and share similar oceanographic characteristics. However, RV001 is more strongly influenced by coastal conditions (Marić et al. 2012; Mozetič et al. 2010). The two stations were selected to characterise the plankton community of the wider coastal zone and to detect possible differences between the stations in terms of plankton community structure (taxonomic and functional diversity). Each sampling was conducted according to an identical schedule, with sampling at the same time (morning, from 8:00 to 10:00) at each station on every sampling cruise.

At each station, biological samples for metatranscriptomics and accompanying environmental data (CTD and nutrient measurements) were collected. Conductivity–temperature–depth (CTD) measurements were taken using an SBE 25 Sea Logger CTD probe (Sea‐Bird Electronics Inc., Bellevue, Washington, USA) deployed from surface to bottom. Nutrient concentrations were measured at three depths (0, 5 and 20 m), and included analyses of nitrate (${\mathrm{NO}}_{3}^{-}$), nitrite (${\mathrm{NO}}_{2}^{-}$), ammonium (${\mathrm{NH}}_{4}^{+}$), orthophosphate (${\mathrm{PO}}_{4}^{3-}$) and orthosilicate (${\mathrm{SiO}}_{4}^{-}$) using spectrophotometric methods as described by Ivančič and Degobbis (1984) and Parsons et al. (1984) on a Shimadzu UV‐1800 spectrophotometer with the method detection limits for (${\mathrm{NO}}_{3}^{-}$), (${\mathrm{NO}}_{2}^{-}$), (${\mathrm{NH}}_{4}^{+}$), (${\mathrm{PO}}_{4}^{3-}$) and (${\mathrm{SiO}}_{4}^{-}$) of 0.05, 0.01, 0.1, 0.02 and 0.05 μmol L^−1^, respectively. Calibration procedures followed the methods described by Strickland and Parsons (1972). Total dissolved P (Total_P) analyses were performed using a chemical combustion method with persulfate (Menzel and Corwin 1965). Dissolved organic P (DOP) was calculated by subtracting concentrations of ${\mathrm{PO}}_{4}^{3-}$ from the Total_P. Dissolved inorganic N (DIN) was calculated as the sum of ${\mathrm{NO}}_{3}^{-}$, ${\mathrm{NO}}_{2}^{-}$ and ${\mathrm{NH}}_{4}^{+}$ concentrations. The nitrate to orthophosphate ratio (${\mathrm{NO}}_{3}^{-}$:${\mathrm{PO}}_{4}^{3-}$) was calculated as the Redfield ratio (N:P = 16:1) as described by Redfield et al. (1963).

Plankton community sampling for metatranscriptomics was carried out using vertical net hauls from a working depth of 25 m to the surface with a phytoplankton net (60‐cm opening diameter, 2‐m length, 50‐μm mesh pore size). Live net samples were immediately filtered onto 1.2‐μm filters, rapidly frozen in liquid nitrogen, and stored at −80°C at the end of each cruise. Total RNA was isolated using the PureLink Mini Kit (Invitrogen), with on‐column DNase treatment (PureLink DNase, Invitrogen), following the manufacturer's instructions and adapted for filter‐based extractions as described in (Knjaz et al. 2024). Total RNA quality checks, library preparation and sequencing were performed by AllGenetics (Spain). RNA samples were quality‐checked using an Agilent 2100 Bioanalyzer (Agilent RNA 6000 Nano Kit) and quantified with the Qubit RNA Assay (Thermo Fisher Scientific). Library preparation was performed using the NEBNext Ultra II Directional RNA Library Prep Kit. Each RNA extract was enriched for mRNA by selecting molecules with a poly‐A tail at their 3′ end using the NEBNext Poly(A) mRNA Magnetic Isolation Module. The captured mRNA molecules were then converted into cDNA, and sequencing adapters were added to their ends. The molecules within each library were indexed with a unique oligonucleotide tag so that the libraries could be pooled for sequencing and demultiplexed after sequencing. The finished libraries were paired‐end sequenced using NovaSeq X Plus 25B (Illumina) sequencing platform and reagents with a 6 Gb output each, containing 150 bp nucleotide fragments.

### 2.3 Bioinformatic Workflow

Raw reads pre‐processing, de novo assembly, open reading frame (ORF) prediction and ORF quantification were conducted as described in Knjaz et al. (2024). Briefly, raw reads were filtered for rRNA using SortMeRNA (v4.3.6) software (Kopylova et al. 2012) with smr_v4.3_default_db.fasta as the reference. Quality trimming was performed using Trimmomatic (v0.39) (Bolger et al. 2014), with settings for the sliding window set to 5:20 and a minimum length of 50 bp. Pre‐processed reads were quality‐checked using FASTQC (v0.11.8) (https://www.bioinformatics.babraham.ac.uk/projects/fastqc/) and MULTIQC (v1.13) (https://github.com/ewels/MultiQC). Trinity software (v2.15.0) (Grabherr et al. 2011) was used for de novo assembly of pre‐processed reads with default settings. ORFs were predicted from assembled transcripts using TransDecoder (v5.7.0) with default settings (https://github.com/TransDecoder/TransDecoder). ORF quantification was performed using Salmon (v1.9.0) (Patro et al. 2017), with indexes constructed for ORFs of each sample separately. ORFs with zero counts were filtered out and excluded from further analysis. Functional annotation of ORFs to KEGG (Kyoto Encyclopedia of Genes and Genomes) (Kanehisa and Goto 2000) was performed using OmicsBox software (OmicsBox—Bioinformatics Made Easy, BioBam Bioinformatics, March 3, 2019, https://www.biobam.com/omicsbox) aiming for KEGG Ortholog (KO) annotations and corresponding KEGG pathways (map). Sequences meeting *E*‐value and bit‐score thresholds of 1 × 10^−5^ and 50, respectively, were considered successfully annotated. Taxonomic annotation of ORFs to the MMETSP dataset (Keeling et al. 2014) was carried out using EUKulele (Krinos et al. 2020) with default settings. Outputs from taxonomic and functional annotations, quantification results, and metadata were merged based on ORF IDs to create a single table for subsequent data analysis and visualisation.

### 2.4 Taxonomic Annotation

The default EUKulele taxonomy for MMETSP was applied. From the annotated phyla, only those containing photosynthetic and mixotrophic representatives were selected for analysis. The aim was to describe the phytoplankton community, as in studies using classical identification methods (microscopy). The selected phytoplankton phyla were Dinoflagellata (with Syndiniales included), Ochrophyta, Haptophyta, Chlorophyta and Cryptophyta. Ochrophyta was further divided into two categories: class Bacillariophyta (diatoms) and Other Ochrophyta, which included the classes Bolidophyceae, Chrysophyceae, Dictyophyceae, Pelagophyceae, Pinguiophyceae, Raphidophyceae, Synchromophyceae, Synurophyceae and Xanthophyceae, to allow for separate discussion. These six groups of interest—Dinoflagellata, Bacillariophyta (diatoms), Other Ochrophyta, Haptophyta, Chlorophyta and Cryptophyta—were considered as representatives of the phytoplankton community. Other phyla, predominantly comprising heterotrophic members of the community, were aggregated into the group ‘Other Eukaryotes’, which included Apicomplexa, Cercozoa, Ciliophora, Discoba, Foraminifera, Glaucophyta, Lobosa, Opalozoa, Rhodophyta, Choanoflagellida, Conosa, Discosea, Fungi, Hacrobia_X, Perkinsea, Stramenopiles_X and Sagenista. ORFs that could not be assigned to any MMETSP phylum were grouped under ‘Unclassified’.

### 2.5 Counts Normalisation

ORF quantification via Salmon (v1.9.0) generated three values: ORF length (length), number of reads mapped to ORF (NumReads), and transcripts per million (TPM), a normalised count value suitable for inter‐sample comparison (Johnson and Krishnan 2022; Zhao et al. 2021). Those TPM values were summed at the phylum level, and cumulative TPM values (TPMSum) were used as a measure of the metabolic activity of targeted phytoplankton taxa within the community (*inter‐phyla normalisation*).

To assess intra‐phylum activity (or activity among taxa of the specific Phylum), TPM normalisations were performed separately for each phylum of interest, allowing for comparisons of metabolic activity within phylum without being influenced by the total community composition (*intra‐phylum normalisation*). We used the following formula (Zhao et al. 2021):

$${\mathrm{TPM}}_{i}=\frac{q_{i}}{l_{i}}\times \frac{1}{\sum_{j}\frac{q_{j}}{l_{j}}}\;\times 10^{6}$$

where *i* denotes ORF of interest an *j* denotes sample. *q* represents the number of reads mapped to ORF *i* and *l* represents ORF length (in kb). The scaling factor, $\sum_{j}\frac{q_{j}}{l_{j}}$, corresponds to the sum of all reads belonging to specific phylum in sample *j*, normalised by ORF length. For this equation ORF length and number of reads mapped to ORF, generated by Salmon, were used. TPM values associated with ORFs sharing identical taxonomic and functional assignments (KO) were summed at the phylum and/or genus level.

### 2.6 Statistical Analysis and Visualisations

#### 2.6.1 Ordination Patterns Analyses

All statistical analyses involving ordinations were conducted using R (R Core Team 2024), and the ‘vegan’ package (Oksanen et al. 2013).

Non‐Metric Multidimensional Scaling (NMDS) based on Bray–Curtis dissimilarity was used to assess the similarity in community functional composition across seasons and stations. Separate analyses were conducted for the overall phytoplankton community and for each targeted phylum individually. For the NMDS analysis of the whole community, inter‐phylum normalised TPM values of KEGG Orthologs (KO) with MMETSP taxonomic annotations belonging to one of the six targeted phyla were summed at the gene level. Similarly, for the NMDS analysis of each phylum separately, intra‐phylum normalised TPM values of KEGG Orthologs (KO) were summed at the gene level. To assess the statistical significance of group differences in community composition, a permutational multivariate analysis of variance (PERMANOVA) was applied. The functions ‘vegdist’ and ‘metaMDS’ were used to compute the Bray–Curtis dissimilarity matrix and perform NMDS, while the ‘adonis’ function was used to perform PERMANOVA.

To explore the relationship between the functional composition of targeted phyla and environmental parameters, a Redundancy Analysis (RDA) was conducted. The environmental variables included concentrations of DIN, Total P and ${\mathrm{SiO}}_{4}^{-}$, as well as temperature and salinity. Mean values for these variables, calculated from measurements at three depths (0, 10 and 20 m), were used in the analysis. To minimise the influence of highly abundant genes, we log_2_ transformed intra‐phylum normalised TPM values prior to analysis. RDA was performed using the ‘rda’ function, and environmental vector fitting was performed using the ‘envfit’ function. The significance of the generated RDA model was evaluated using permutation‐based ANOVA with the ‘anova’ function, identifying key environmental drivers.

#### 2.6.2 Genes and Pathways Expression Patterns

As dinoflagellates were the most dominant group in terms of metabolic activity in our dataset, we selected the phylum Dinoflagellata for further discussion of KEGG Orthologs (KO) and annual pathway expression patterns.

To assess gene expression levels, we calculated the mean, variance and quartiles of intra‐phylum TPM values for uniquely annotated KEGG Orthologs (KO). The 60 genes with the highest mean intra‐phylum TPM normalised values were selected for further interpretation, along with additional genes of interest related to nitrogen and phosphorus acquisition and utilisation identified in the literature. The annual expression patterns of these genes were visualised using heatmaps generated with the ‘pheatmap’ package (Kolde 2019) in R. Hierarchical clustering was applied to group genes with similar transcriptional activity, using Euclidean distance as the similarity metric.

To explore the overall community metabolic profile, functionally annotated KEGG Orthologs (KO) were assigned to their corresponding pathways, and intra‐phylum TPM normalised gene expression levels were aggregated based on their KEGG pathway affiliation. We selected pathways from four categories considered relevant for interpreting eukaryotic phytoplankton functional succession: Metabolism, Genetic Information Processing, Environmental Information Processing and Cellular Processes (https://www.genome.jp/kegg/pathway.html). The annual expression patterns of selected pathways were visualised using heatmaps generated with the pheatmap package (Kolde 2019) in R with no clustering applied.

## 3 Results

### 3.1 Environmental Parameters

**FIGURE 2:**
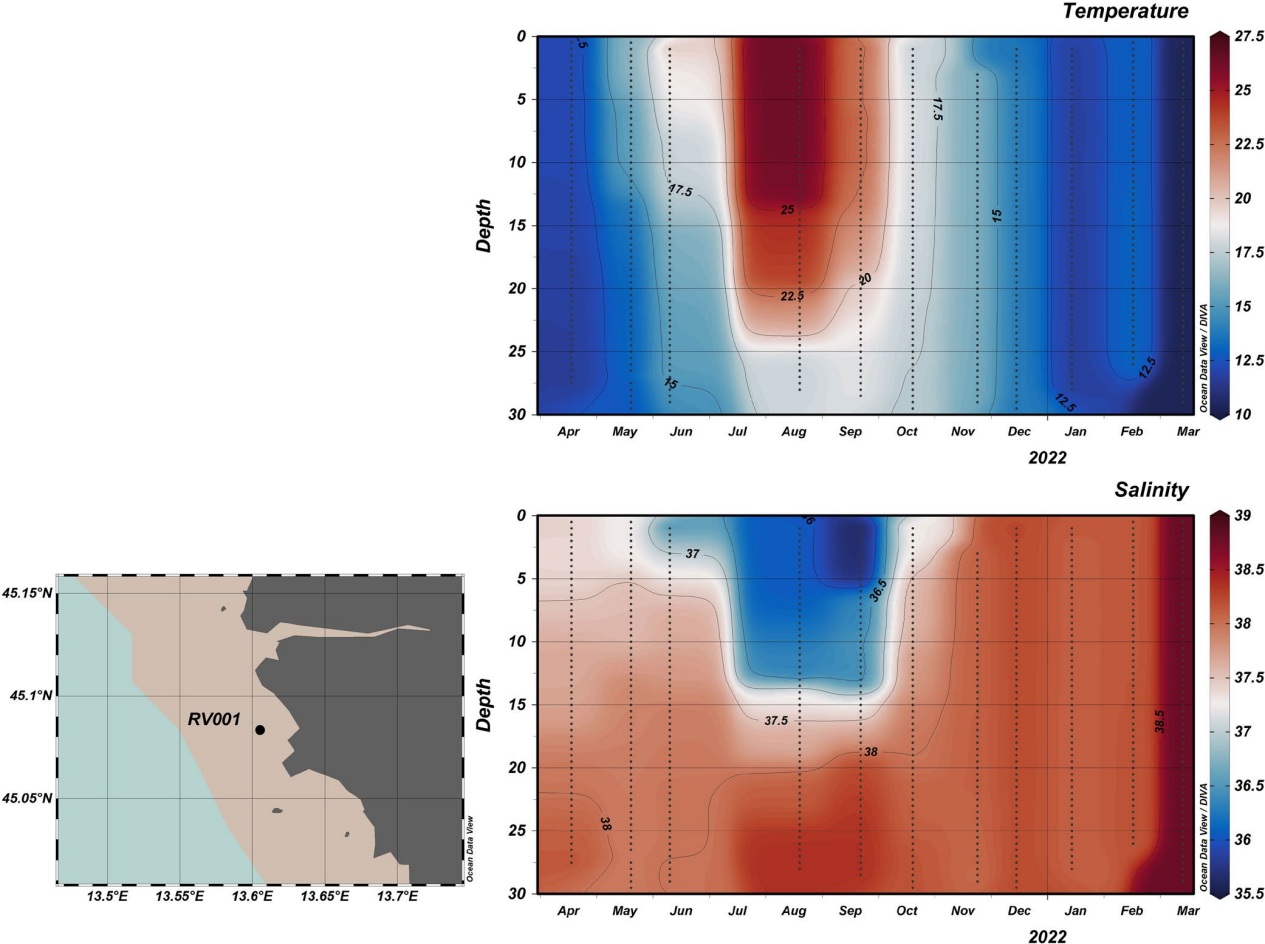
Vertical sections (°C) of temperature and salinity on station RV001, plotted using DIVA interpolation in Ocean Data View.

**FIGURE 3:**
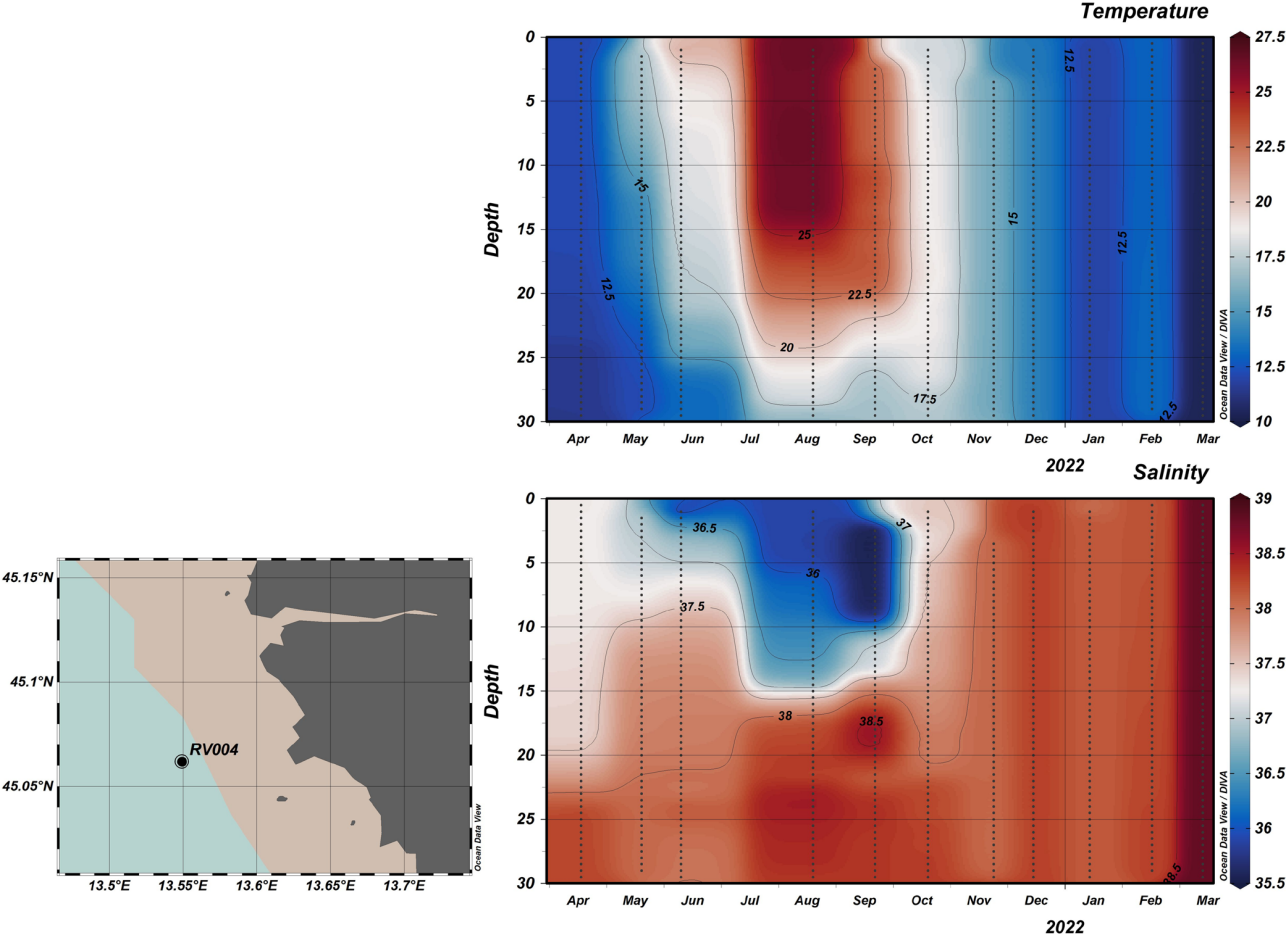
Vertical sections (°C) of temperature and salinity on station RV004, plotted using DIVA interpolation in Ocean Data View.

**FIGURE 4:**
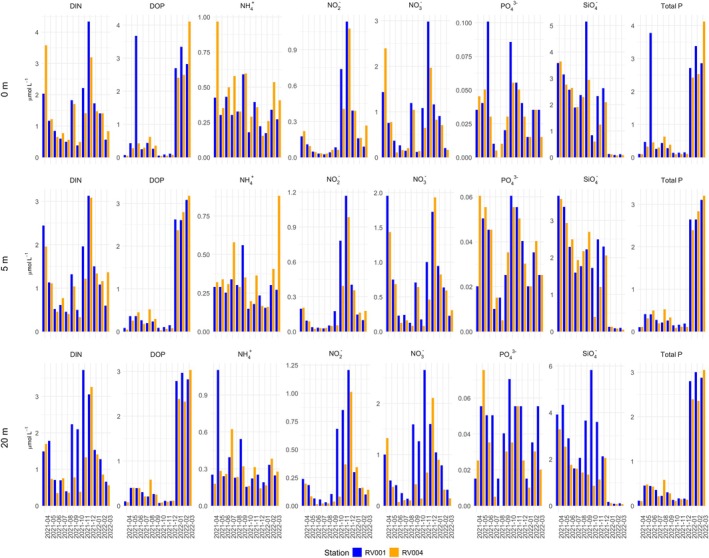
Annual distribution nutrient concentrations on stations RV001 and RV004 on 12 timepoints and three depths (0, 5 and 20 m). All concentrations are reported in μmol L^−1^. ${\mathrm{NO}}_{3}^{-}$ (nitrate), ${\mathrm{NO}}_{2}^{-}$ (nitrite), ${\mathrm{NH}}_{4}^{+}$ (ammonium), DIN (dissolved inorganic N (sum of (${\mathrm{NO}}_{3}^{-}$), (${\mathrm{NO}}_{2}^{-}$) and (${\mathrm{NH}}_{4}^{+}$))), ${\mathrm{PO}}_{4}^{3-}$ (orthophosphate), ${\mathrm{SiO}}_{4}^{-}$ (orthosilicate), Total_P (total dissolved P), DOP (dissolved organic P (sum of Total_P and (${\mathrm{PO}}_{4}^{3-}$))).

**FIGURE 5:**
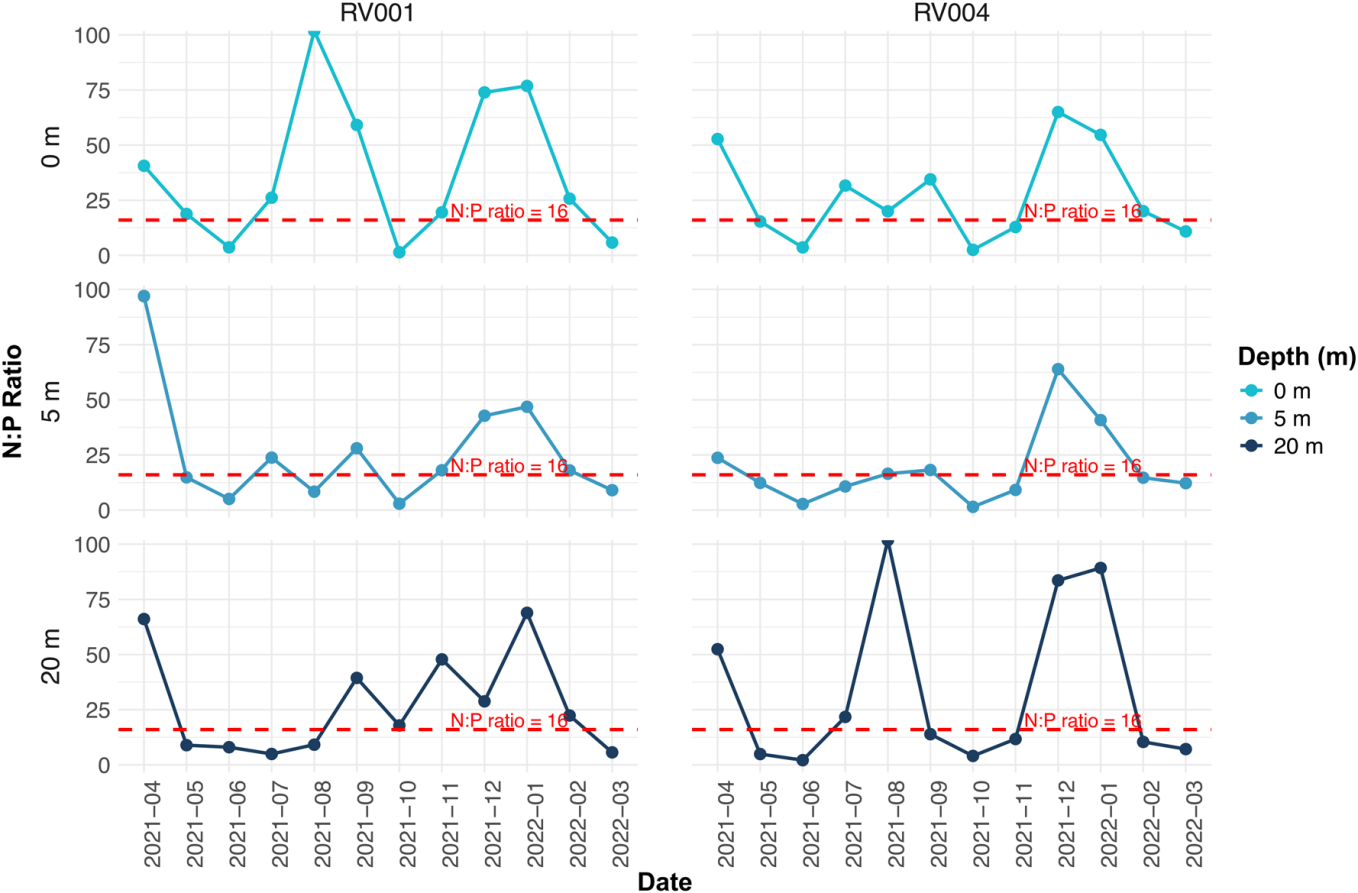
Annual distribution of N:P ratio (nitrate (${\mathrm{NO}}_{3}^{-}$)/ orthophosphate (${\mathrm{PO}}_{4}^{3-}$)). Red dashed line depicts N:P ratio = 16.

During the 1‐year sampling period, which covered all four seasons, temperatures ranged from 10.4°C to 26.5°C. In summer, high temperatures persisted throughout most of the water column, but there was a difference of around 10°C between the surface and bottom (30 m) layers, indicating the presence of stratification (Figures 2 and 3). Salinity ranged from 35.8 to 37.9. From late spring through summer, a noticeable drop in surface water salinity was observed (Figures 2 and 3), contributing to water column stratification. A peak in Total P was recorded in surface waters at station RV001 in June (Figure 4), when both ${\mathrm{PO}}_{4}^{3-}$ and DOP reached high concentrations of 0.1 and 3.7 μmol/L, respectively. For the rest of the summer, Total P and dissolved DIN were almost depleted throughout the water column at both stations (Figure 4). Nutrient concentrations began to regenerate in autumn (Figure 4). DIN concentrations peaked in November and December at both stations (> 3 μmol/L). ${\mathrm{SiO}}_{4}^{-}$ concentrations were higher during the first part of the year (Figure 4), with the highest concentrations recorded at station RV001: 5.2 μmol/L in September and 5.8 μmol/L in October. The concentrations then decreased, reaching < 0.2 μmol/L in winter. During the winter months, DOP concentrations were higher compared with the rest of the year (Figure 5), ranging from 2.3 to 4.1 μmol/L. The N:P ratio was unbalanced in favour of NO_3_^−^ (N:P > 16) for most of the year (Figure 5). At both stations and at all three measured depths, the N:P ratio was < 16 only in June and March (Figure 5). At station RV004, an N:P ratio < 16 was also recorded at all three measured depths in May, October and November (Figure 5).

### 3.2 General Overview of the Dataset

The paired‐end Illumina sequencing generated 957,107,872 raw reads for 24 libraries, representing 24 samples collected from two stations (RV001 and RV004) each month from April 2021 to March 2022. Trinity assembly yielded a total of 12,603,917 transcripts, from which 4,872,492 protein sequences were predicted. After eliminating protein sequences with zero counts using Salmon software, 4,609,173 sequences remained. Using EUKulele software, 45.6% of sequences across the entire dataset were successfully annotated at the phylum level, totalling 2,102,068 sequences. Taxonomic annotation identified 22 different eukaryotic phyla. The photosynthetic and mixotrophic protist phyla—Dinoflagellata, Ochrophyta (divided into Bacillariophyta and Other Ochrophyta), Haptophyta, Chlorophyta and Cryptophyta—were selected to represent the phytoplankton community. Other eukaryotic phyla (grouped as ‘Other Eukaryotes’) found in the dataset included Apicomplexa, Cercozoa, Ciliophora, Discoba, Foraminifera, Glaucophyta, Lobosa, Opalozoa, Rhodophyta, Choanoflagellida, Conosa, Discosea, Fungi, Hacrobia_X, Perkinsea, Stramenopiles_X and Sagenista. The dataset of six phytoplankton community phyla selected for further analyses comprised 1,059,949 protein sequences, of which 837,676 were successfully functionally annotated with KEGG Ortholog (KO) annotations. There were 7765 unique orthologues. The highest number of unique KEGG Ortholog (KO) annotations (genes) was recorded for Dinoflagellata (6534), followed by Chlorophyta (4301). All other phyla had fewer than 4000 unique genes, with Bacillariophyta having the smallest number of orthologues (3189). Overall, 1200 genes were shared among the six phytoplankton phyla.

### 3.3 Taxonomic Succession of the Phytoplankton Community Metabolic Activity

**FIGURE 6:**
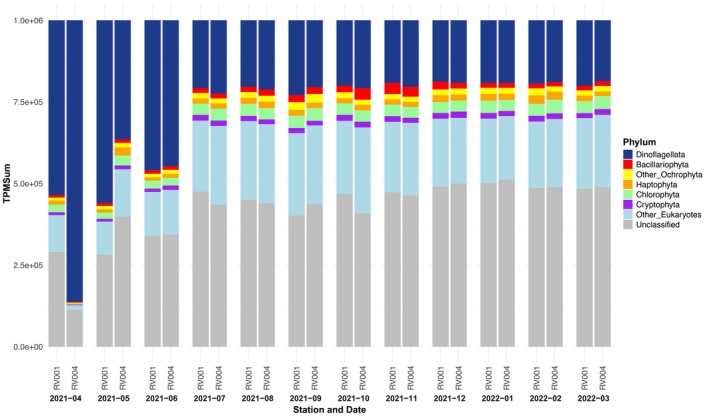
Annual phyla metabolic activity, based on inter‐phylum TPM normalised values. *X*‐axis represents stations (RV001 and RV004) and 12 sampling timepoints. *Y*‐axis represents cumulative inter‐phylum TPM normalised values, summed by phylum taxonomic level.

Analysis of metabolic activity, based on inter‐phylum TPM normalised values summed by taxonomic level, revealed year‐round dominance of dinoflagellate activity within phytoplankton in the size fraction greater than 50 μm (Figure 6). Their relative expression throughout the year showed a similar pattern at both sampling stations and was nearly equal to the relative expression of the category ‘other Eukaryotes’, which accounted for the highest proportion of taxonomically annotated ORFs (Figure 6).

**FIGURE 7:**
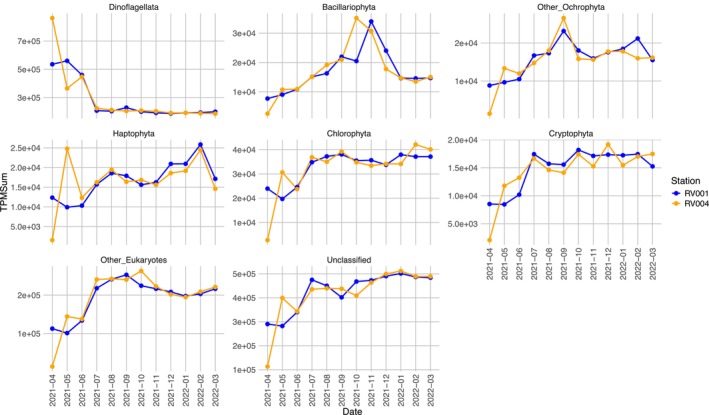
Annual phyla metabolic activity, based on inter‐phylum TPM normalised values, displayed on a separate scale to highlight elevated activity levels for phyla. *X*‐axis represents stations (RV001 and RV004) at 12 sampling timepoints. *Y*‐axis represents cumulative inter‐phylum TPM normalised values, summed by phylum taxonomic level.

**FIGURE 8:**
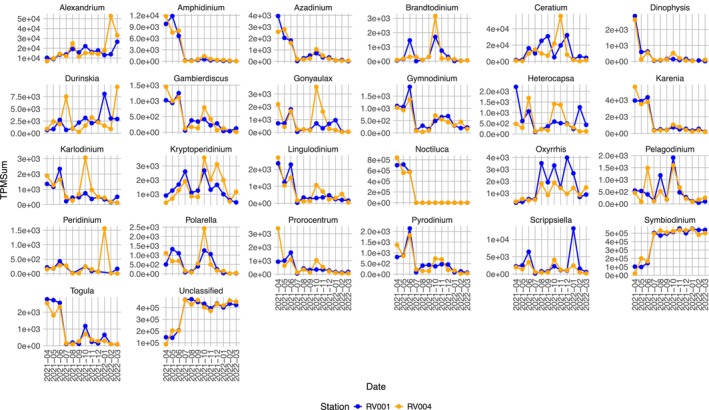
Dinoflagellate genera metabolic activity, based on intra‐phylum TPM normalised values. *X*‐axis represents stations (RV001 and RV004) at 12 sampling timepoints. *Y*‐axis represents cumulative inter‐phylum TPM normalised values, summed by genus taxonomic level. Each taxon is displayed on a separate scale to highlight elevated activity levels for the genera. ‘Unclassified’ refers to reads assigned to Dinoflagellata that could not be further classified to a specific genus.

In spring, dinoflagellate expression accounted for a significant proportion of taxonomically annotated ORFs, exceeding at the phylum level even that of ‘other Eukaryotes’ and ‘unclassified’ (Figures 6 and 7). A closer examination of dinoflagellate expression at the genus level highlighted the *Noctiluca* genus as having particularly high transcription activity, with a peak at station RV004 in April (TPMSum > 800,000) (Figure 8). *Noctiluca*‐assigned ORF TPMSum values declined throughout the rest of spring, reaching their lowest in July (Figure 8). The *Noctiluca* TPMSum peak coincided with elevated TPMSum values (compared with the rest of the year) from several other dinoflagellate genera, including *Amphidinium* (TPMSum > 12,000), *Azadinium* (TPMSum > 4000), *Dinophysis* (TPMSum > 2500), *Gambierdiscus* (TPMSum > 1500), *Gymnodinium* (TPMSum > 2000), *Heterocapsa* (TPMSum > 2000), *Karenia* (TPMSum > 5000), *Lingulodinium* (TPMSum > 2500), *Prorocentrum* (TPMSum > 3000) and *Togula* (TPMSum > 3000) (Figure 8). Throughout the rest of the year, dinoflagellate‐assigned ORFs continued to dominate the phytoplankton community, with relatively stable expression levels overall (Figures 6 and 7).

All other recorded phyla of the phytoplankton community (Bacillariophyta, other Ochrophyta, Haptophyta, Chlorophyta and Cryptophyta) had the lowest relative expression values during the spring months at both stations (Figure 7). As an exception, the phylum Haptophyta showed higher values in May at station RV004, when high spring *Noctiluca* activity was still evident at both sampling stations (Figure 7).

The composition of dinoflagellate genera contributing to the overall high phylum activity in the dataset differed in spring compared with the rest of the year (Figures 7 and 8). Notably, the genus *Symbiodinium*, a group including endosymbiotic dinoflagellates, exhibited a rise in TPMSum values from July onwards (Figure 8). The high activity of *Symbiodinium* (TPMSum > 500,000) persisted throughout the rest of the year, with stable transcription levels at both stations (Figure 8). In the annual succession of phytoplankton expression, some other dinoflagellate genera also exhibited peaks in activity. Generally, the two sampling stations shared similar expression patterns for all detected dinoflagellate genera, with some exceptions. For instance, at station RV004, *Ceratium* showed several distinct peaks of activity during late summer (TPMSum > 30,000) and autumn (TPMSum > 50,000), while *Alexandrium* exhibited its highest peak in late winter (TPMSum > 50,000) (Figure 8). Similarly, expression of *Karlodinium*, *Gonyaulax* and *Peridinium* was characterised by peaks only at station RV004 during autumn and winter.

Chlorophyta and Cryptophyta TPMSum values also showed higher values in summer, after the *Noctiluca* bloom ended (Figure 7). Activities of these phyla remained high and relatively stable throughout the rest of the year (Figure 7). Other Ochrophytes and Bacillariophyta (diatoms) gradually increased transcription activity during summer (Figure 7). Other Ochrophytes had the most distinct TPMSum value peak in late summer at both stations (Figure 7). At both stations, a peak of Bacillariophyta activity was detected in autumn (Figure 7). The Bacillariophyta activity peak first occurred at RV004 in October and persisted during November, while at RV001 the activity peak appeared only in November (Figure 7). During winter, the activity of other Ochrophytes and Bacillariophyta (diatoms) decreased compared with autumn, but still remained higher than in spring (Figure 7). The haptophytes had their highest activity peaks in the winter months, reaching the highest TPMSum values in February at both stations (Figure 7).

### 3.4 Phytoplankton Community Ordination Patterns

**FIGURE 9:**
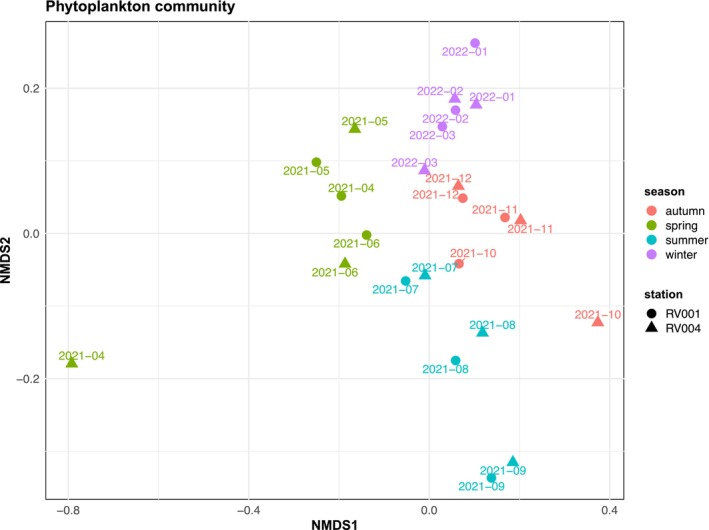
NMDS results of overall phytoplankton community functional composition based on Bray–Curtis dissimilarity. Points represent samples coloured by season and shaped by sampling station. Labels above points represent sampling timepoint.

NMDS analysis of the 1‐year metatranscriptome dataset revealed a seasonal pattern in sample grouping, based on the functional composition and expression of the phytoplankton community, with 43% of the variance (*R* ^2^ = 0.43, *F* = 5.05) among samples explained by the season parameter (Pr(>*F*) = 0.001) (Figure 9, Table S1). The station parameter had a limited effect on sample ordination (*R* ^2^ = 0.02, Pr(>*F*) = 0.999) (Figure 9, Table S2). Spring, autumn and winter samples formed a relatively strong cluster, with only samples from station RV004 (April and October) separated from the other spring and autumn samples, respectively (Figure 9).

**FIGURE 10:**
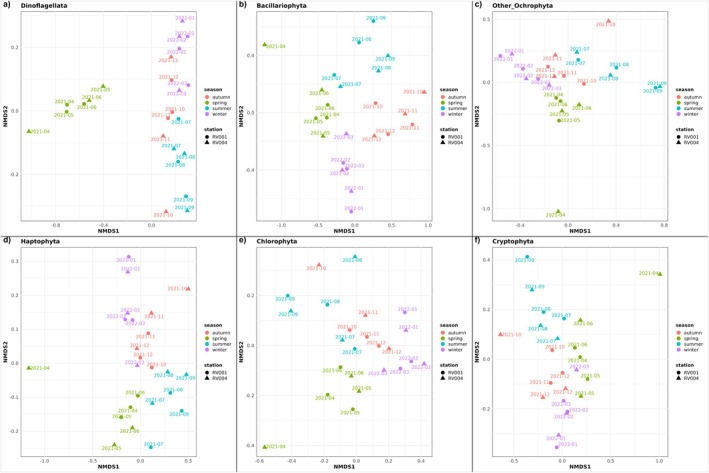
Phylum‐specific NMDS results of functional composition based on Bray–Curtis dissimilarity. Panels (a–f) represent different phytoplankton phyla: (a) Dinoflagellata, (b) Bacillariophyta, (c) Other Ochrophyta, (d) Haptophyta, (e) Chlorophyta and (f) Cryptophyta. Points represent samples coloured by season and shaped by sampling station. Labels above points represent sampling timepoint.

Phylum‐specific NMDS analyses also showed distinct seasonal ordination for all phytoplankton phyla (Pr(>*F*) < 0.05), while the station parameter had limited influence on sample ordination (Pr(>*F*) > 0.05) (Figure 10, Tables S1 and S2). The proportion of variance explained by the season parameter ranged from 33% to 73% (Table S1). Dinoflagellates had the highest proportion of variance explained (*R* ^2^ = 0.73, *F* = 18.13), followed by Bacillariophyta (*R* ^2^ = 0.43, *F* = 5.92) (Table S1). All other phyla had less than 35% of variance explained by the season parameter (*R* ^2^ < 0.35, *F* < 4) (Table S1). For all phyla, samples from station RV004 (April and October) were distant from their respective season groups (Figure 10).

**FIGURE 11:**
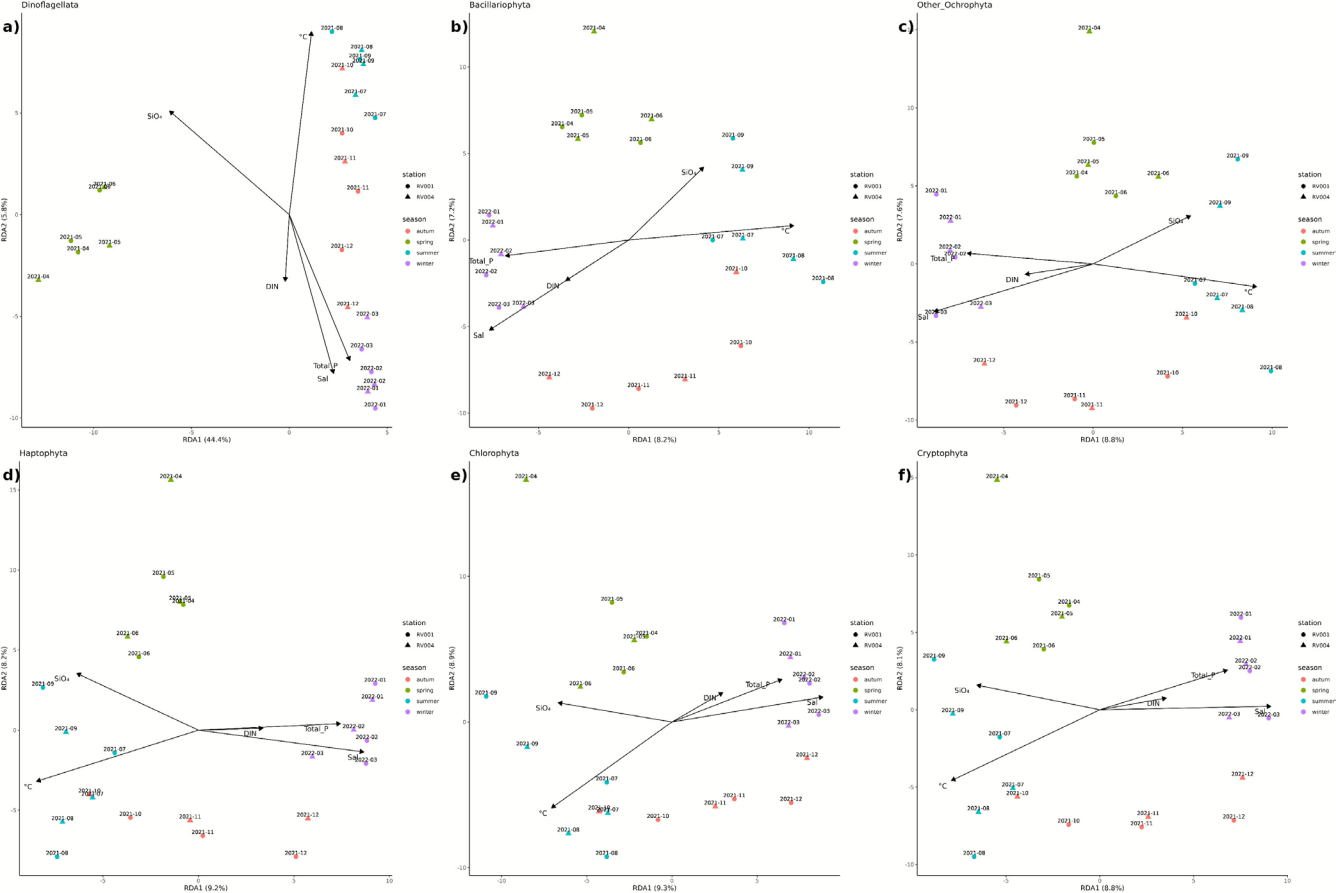
Redundancy Analysis (RDA) plots showing the relationships between environmental variables and phytoplankton community composition at the phylum level. Panels (a–f) represent different phytoplankton phyla: (a) Dinoflagellata, (b) Bacillariophyta, (c) Other Ochrophyta, (d) Haptophyta, (e) Chlorophyta and (f) Cryptophyta. Points indicate samples, coloured by season and shaped by sampling station. Labels above points represent sampling timepoints. Arrows indicate environmental variables influencing community composition, including temperature (°C), dissolved inorganic nitrogen concentration (DIN), orthosilicate concentration (${\mathrm{SiO}}_{4}^{-}$), salinity (Sal) and total phosphorus concentration (Total_P). The length and direction of the arrows represent the strength and correlation of each variable with the RDA axes. Percentages in brackets indicate the portion of variance explained by RDA1 and RDA2 axes.

The results of the RDA (Redundancy Analysis) models indicate that environmental or explanatory variables significantly influenced the functional composition of all examined phytoplankton phyla (Pr(>*F*) = 0.001) (Figure 11, Table S3). Among these, Dinoflagellata exhibited the strongest response to the model, with the highest explained variance (*F* = 4.8216) (Table S3). Bacillariophyta, Other Ochrophyta, Haptophyta, Chlorophyta and Cryptophyta also showed significant but comparatively lower *F*‐values (ranging from 1.4731 to 1.6808) (Figure 11, Table S3). All studied environmental variables had significant effects on the RDA models for all phyla except for DIN (Pr(>*F*) > 0.05) (Table S4). ${\mathrm{SiO}}_{4}^{-}$ was the most consistently significant factor (Pr(>*F*) ≤ 0.001) across all phyla (Figure 11, Table S4). Total dissolved phosphate and temperature also had significant effects on all phyla (Pr(>*F*) < 0.05) (Figure 11, Table S4). Salinity significantly affected all phyla (Pr(>*F*) < 0.05), except Dinoflagellata (Pr(>*F*) = 0.07) (Figure 11, Table S4). In all RDA models, the vectors representing dissolved inorganic nitrogen, total dissolved phosphate and salinity were oriented in the same direction, aligning with winter samples. In contrast, the vector for ${\mathrm{SiO}}_{4}^{-}$ was oriented oppositely, towards late summer samples, while the temperature vector was directed towards summer samples. Notably, no environmental variables exhibited strong directional alignment with spring or autumn samples.

### 3.5 Functional Succession of Dinoflagellate Metabolic Activity

#### 3.5.1 Pathways Expression Patterns

**FIGURE 12:**
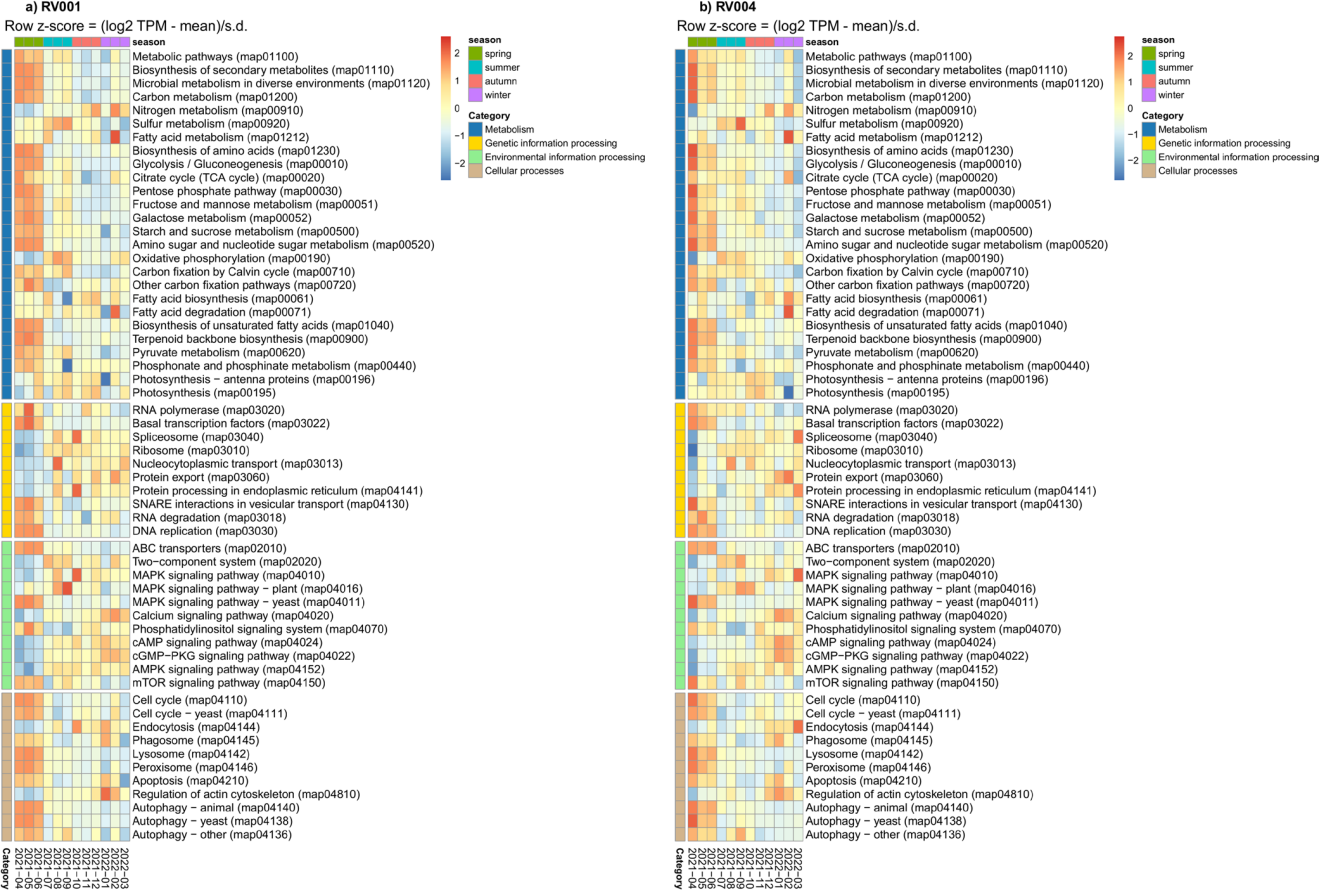
Annual succession of pathways utilised by phylum Dinoflagellata visualised by station RV001 (left) and RV004 (right). Column labels indicate 12 sampling points. Column annotations indicate season. Row annotation indicate four pathway categories: metabolism, genetic information processing, environmental information processing and cellular processes. Expression values on pheatmaps are represented as the *z*‐score (log_2_(TPM) – mean (row)/SD (row)).

Since all sampling was conducted at the same time of day, in the morning, the effect of phytoplankton diurnal metabolic cycles on the observed gene and pathway expression patterns was minimised, allowing the observed expression patterns to be interpreted in the context of seasonal succession. The pathways utilised by dinoflagellates showed, at both stations, distinct fingerprints in spring, during *Noctiluca* dominance, compared with the rest of the year (Figure 12). Most pathways within the Metabolism and Cellular Processes categories exhibited higher expression levels during spring than during the rest of the year. Among the Metabolism pathways, those related to energy acquisition, such as glycolysis/gluconeogenesis (ko0010), the TCA cycle (ko00020) and pyruvate metabolism (ko00620), showed elevated expression. Additionally, carbohydrate metabolism pathways, such as fructose and mannose metabolism (ko00051), galactose metabolism (ko00052) and starch and sucrose metabolism (ko00500), were also highly expressed. The carbon fixation by the Calvin cycle (ko00710) pathway, which primarily includes genes involved in the dark reactions of photosynthesis, exhibited high expression levels during spring and continued through summer. In contrast, pathways associated with the light‐dependent reactions of photosynthesis (ko00195) and photosynthesis–antenna proteins (ko00196) showed the lowest expression levels during spring and peaked during summer and autumn. The oxidative phosphorylation pathway (ko00190) had the highest expression levels during summer, with a slightly less pronounced increase in late winter (Figure 12).

Meanwhile, the phosphonate and phosphinate metabolism pathway (ko00440) showed increased expression in spring (Figure 12), whereas sulphur (ko00920) and nitrogen (ko00910) metabolism were more pronounced in summer and winter, respectively. Within Cellular Processes, pathways related to cell proliferation, such as cell cycle (ko04110) and cell cycle–yeast (ko04111), had the highest expression levels in spring. These were accompanied by cellular processes pathways involved in heterotrophic feeding, including phagosome (ko04145), lysosome (ko04142) and peroxisome (ko04146), as well as apoptosis (ko04210) and autophagy (animal (ko04140), yeast (ko04138) and other (ko04136)). However, phagosome expression remained high in winter, along with other pathways involved in heterotrophic feeding, such as endocytosis (ko04144) and regulation of the actin cytoskeleton (ko04810).

Pathways involved in genetic information processing also showed seasonal variation. In spring, RNA polymerase (ko03020), basal transcription factors (ko03022), SNARE interactions in vesicular transport (ko04130), RNA degradation (ko03018) and DNA replication (ko03030) pathways were highly transcribed. In contrast, during the rest of the year, pathways such as spliceosome (ko03040), ribosome (ko03010), nucleocytoplasmic transport (ko03013), protein export (ko03060) and protein processing in the endoplasmic reticulum (ko04141) showed increased transcription (Figure 12).

Similarly, pathways involved in environmental information processing exhibited distinct seasonal patterns. In spring, most pathways in this category had their lowest expression levels, except for ABC transporters (ko02010), MAPK signalling pathway—yeast (ko04011), phosphatidylinositol signalling system (ko04070) and mTOR signalling pathway (ko04150), which displayed elevated transcription. All other environmental information processing pathways were transcribed at higher levels during the remainder of the year. The two‐component system (ko02020), MAPK signalling pathway (ko04010) and MAPK signalling pathway—plant (ko04016) were highly transcribed during summer and early autumn. In late autumn and winter, the most highly transcribed pathways were mainly calcium (ko04020), cAMP (ko04024) and cGMP‐PKG (ko04022) signalling (Figure 12).

#### 3.5.2 Gene Transcription Patterns

**FIGURE 13:**
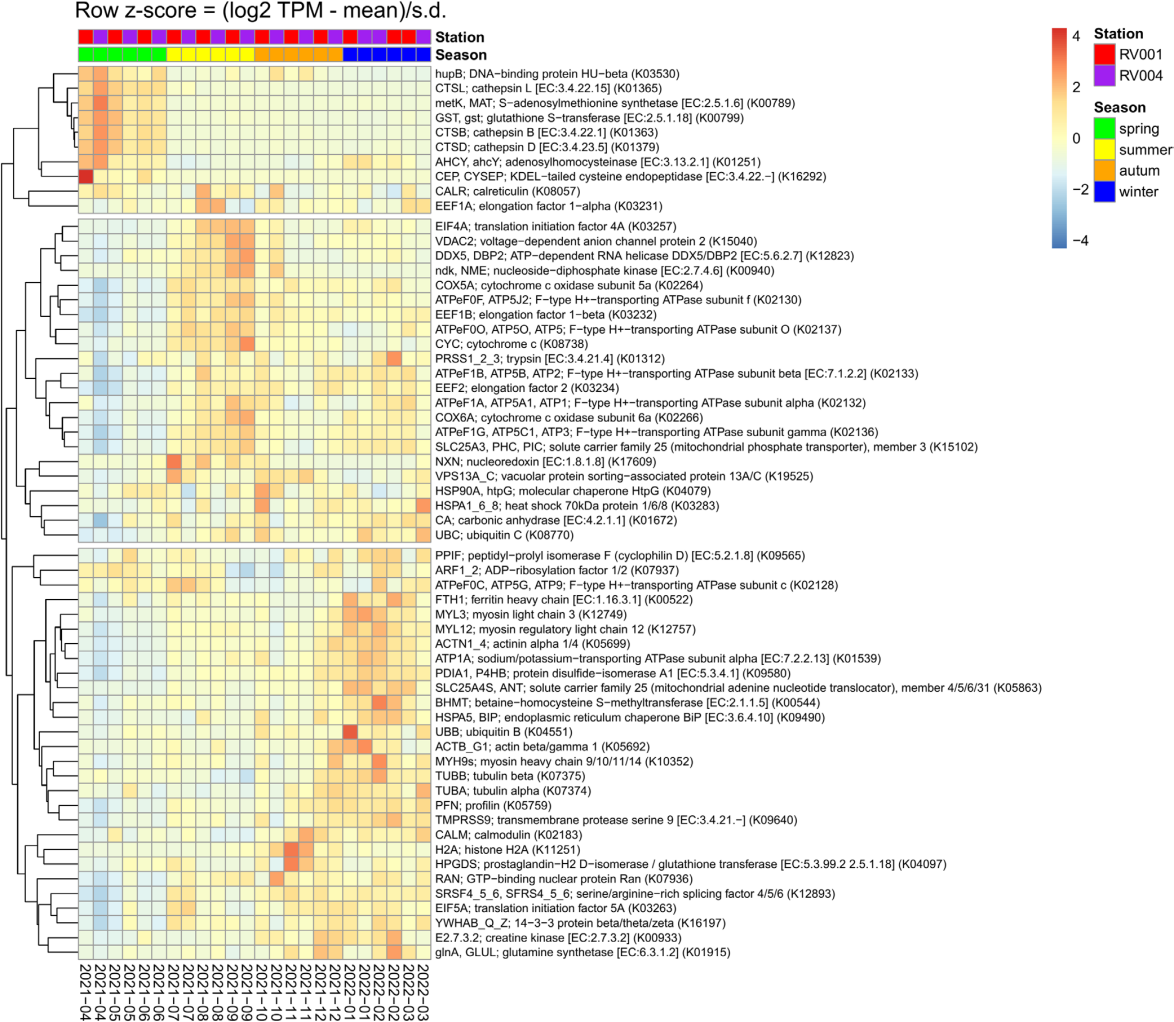
Annual succession of annual succession of 60 genes with the highest mean intra‐phylum TPM normalised values, utilised by phylum Dinoflagellata. Column labels indicate 12 sampling points. Column annotations indicate sampling date, station and season. Row annotation indicate categories: nitrogen and phosphorous assimilation and utilisation. Expression values are represented as the *z*‐score (log_2_(TPM) – mean (row)/SD (row)).

Similarity analysis of the 60 genes with the highest mean intra‐phylum TPM normalised values revealed three distinct clusters based on similar expression patterns (Figure 13). The first cluster comprised genes highly expressed in spring, while the second cluster included genes with elevated expression in summer and early autumn. The third cluster consisted of genes predominantly expressed in late autumn and winter. No differences between the two stations were detected for the observed seasonal variations in the most highly expressed dinoflagellate genes. In the spring cluster, high transcription of genes involved in phagotrophic feeding was observed. These were three cathepsin‐coding genes (L [K01365], B [K01363] and D [K01379]), KDEL‐tailed cysteine endopeptidase (K16292) and calreticulin (K08057). The second cluster was characterised by high transcription levels of genes involved in oxidative phosphorylation: ATP synthase subunits (F‐type ATPase, K02130, K02132, K02133, K02136), cytochrome c oxidase subunits (K02264, K02266) and electron transport components, including cytochrome *c* (K08738) and voltage‐dependent anion channel protein 2 (K15040). The third cluster contained the largest number of genes (28) and appeared to be the most diverse in terms of gene functions. Notably, in this cluster, high expression of cytoskeletal components was found in late autumn and winter. These were mainly actin (K05699 and K05692), tubulin (K07375 and K07374) and myosin (K12749, K12757 and K10352) related genes. No core photosynthesis genes were found among the 60 genes with the highest mean intra‐phylum TPM normalised values. However, α‐carbonic anhydrase (K01672), involved in carbon‐concentrating mechanisms (CCM), was identified and showed elevated transcription during the rest of the year compared with spring. Additionally, calmodulin (K02183), a gene involved in several environmental processing pathways, exhibited higher transcription in late autumn and winter.

**FIGURE 14:**
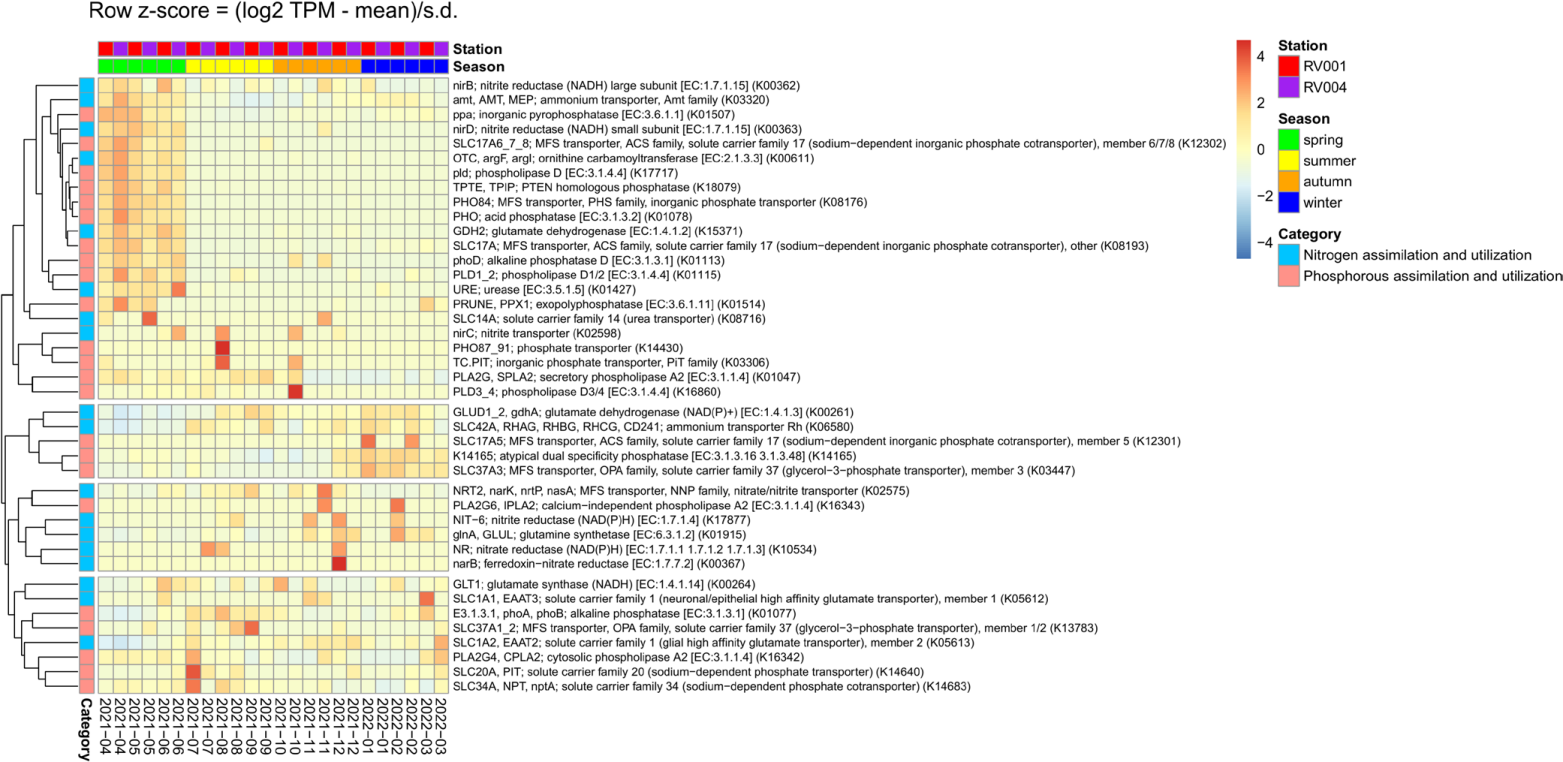
Annual succession of genes involved in nitrogen and phosphorous metabolism utilised by phylum Dinoflagellata. Column labels indicate 12 sampling points. Column annotations indicate sampling date, station and season. Row annotation indicate categories: nitrogen and phosphorous assimilation and utilisation. Expression values are represented as the *z*‐score (log_2_(TPM) – mean (row)/SD (row)).

The analysis of genes involved in nitrogen and phosphorus metabolism revealed four distinct clusters based on similar expression patterns (Figure 14). The first cluster comprised genes highly transcribed in spring, while the second cluster included genes with elevated transcription in late autumn and winter. The third and fourth clusters consisted of genes transcribed in summer, autumn and winter. The transcription patterns of key genes involved in nitrogen and phosphorus utilisation were particularly distinct in spring compared with the rest of the year. For example, no nitrate or nitrite transporters showed high expression levels during this period, whereas the Amt family ammonium transporter (K03320) and urease (K01427) were highly transcribed throughout spring. There was also consistently high expression of the two nitrite reductase genes nirB (K00362) and nirD (K00363) during this period. Additionally, three genes from the MFS family displayed consistently high transcription levels during spring: the high‐affinity inorganic phosphate transporter (PHO84, K08176) and two low‐affinity phosphate transporters (subfamily SLC17A, K12302 and K08193). Several phosphatase‐coding genes were also highly transcribed, involved in alternative phosphorus acquisition strategies from organic sources. These included phosphatase D (phoD, K01113), PTEN homologous phosphatase (K18079) and acid phosphatase (PHO, K01078), which plays a role in hydrolysing organic phosphorus compounds. Moreover, genes involved in phosphorus recycling, such as exopolyphosphatase (PPX1, K01514), inorganic pyrophosphatase (PPA, K01507) and phospholipases D (pld, K17717) and PLD1/2 (K0115), also exhibited high transcription levels during spring.

During summer, after the termination of the *Noctiluca* bloom, several genes involved in nitrogen and phosphate metabolism exhibited elevated transcription in some samples, including high‐ and low‐affinity phosphate transporters (PHO87_91, TC.PIT, SLC20A and SLC34A), alkaline phosphatase (phoA, phoB, K01077), phospholipases (K01047, K16860, K16324), as well as nitrite transporters (nirC). During late autumn and winter, more genes involved in nitrogen assimilation and utilisation had higher transcription values than in the rest of the year. These were, for example, nitrate reductase (K10534) and ferredoxin‐nitrate reductase (K00367) in December as well as nitrite reductase (NAD(P)H) (K17877) in November, December and February. Several genes involved in the assimilation of nitrogen into amino acids also showed elevated expression, such as glutamine synthetase (K01915), glutamate synthase (K00264) and glutamate dehydrogenase (K00261). Throughout winter, an atypical dual‐specificity phosphatase (K14165) and a phosphate transporter from the MFS family (K03447) had consistently higher transcription levels.

## 4 Discussion

### 4.1 Annual Succession—Patterns, Similarities and Differences With Previous Studies

The annual phytoplankton (size fraction greater than 50 μm) succession determined using a metatranscriptomic approach both aligns with and diverges from previous studies. Diatoms (Bacillariophyta) have been considered the most important contributors to overall phytoplankton abundance in the northern Adriatic, with dinoflagellate populations increasing mainly during summer (Aubry et al. 2004, 2012; Godrijan et al. 2013; Marić et al. 2012). In recent years, however, several incidents have supported the notion of a trend towards an increased contribution of dinoflagellates to the phytoplankton community of the north‐eastern Adriatic (Vlašiček et al. 2025). Our findings highlight dinoflagellates as the most prominent phytoplankton group, in the size fraction greater than 50 μm, throughout 2021 when considering metabolic activity. During spring 2021, the abundance of the genus *Noctiluca* aligns with our previous findings based on metabarcoding (Grižančić et al. 2023). It is not surprising that *Noctiluca* accounts for a significant portion of annotated open reading frames (ORFs) during this period, even exceeding those of heterotrophic groups. *Noctiluca* is a well‐known heterotroph capable of feeding on a wide variety of organisms, including both phototrophs and other heterotrophs (Mikaelyan et al. 2014), which suggests that *Noctiluca* predation significantly reduced the abundances of other eukaryotic phyla. Our results indicate that a significant, if not dominant, part of the biological activity in the spring phytoplankton community can be attributed to the genus *Noctiluca*. Interestingly, along with other dinoflagellate genera (*Amphidinium*, *Azadinium* and *Dinophysis*) that coincided with *Noctiluca's* spring dominance, relatively high activity of *Gambierdiscus* was also recorded. This typically benthic genus is considered widely distributed throughout tropical and subtropical regions (Litaker et al. 2009) of the world but occasionally recorded also in temperate waters, including Mediterranean (Aligizaki and Nikolaidis 2008) but never in the Adriatic Sea. Since members of this genus are notorious for producing a group of toxins that can accumulate in benthic‐feeding organisms and be transferred along the food chain (Chateau‐Degat et al. 2005), detected *Gambierdiscus* activity represents a finding of great importance for environmental health. However, to evaluate in more detail the physiological patterns of *Gambierdiscus* potentially present in the Adriatic, further and more extensive research on this genus, including its in situ morphological conformation, is needed. Following the termination of the *Noctiluca* bloom, we observed a rise in the TPMSum values for the genus *Symbiodinium*, which accounted for nearly half of the dinoflagellate‐assigned ORF counts. This photosynthetic dinoflagellate genus establishes mutualistic symbioses with a wide diversity of benthic hosts (Djeghri et al. 2019; Huang et al. 2013; Stat et al. 2008) but its pelagic endosymbioses have also been recorded (Decelle et al. 2018; Mordret et al. 2016). In general, knowledge on free‐living *Symbiodinium* presence and metabolic importance in the environment (ex hospite) is scarce. Recently, metabarcoding and metatranscriptomics studies report on *Symbiodinium* worldwide occurrence and activity in the pelagic realm (Decelle et al. 2018) and high diversity of free‐living *Symbiodinium* species compared with benthic endosymbionts (Huang et al. 2013). Similarly, for the northern Adriatic, only recent metabarcoding detection of the *Symbiodinium* confirmed this dinoflagellate genus pelagic presence (Grižančić et al. 2023) but the role of *Symbiodinium* in the northern Adriatic ecosystem functioning is completely unexplored, giving the even greater significance of the present study recorded high *Symbiodinium* metabolic activity during 2021 dinoflagellate dominance. Phytoplankton net vertical hauls were conducted for community sampling, which biases towards larger cells and excludes the possibility of free‐living *Symbiodinium* being present in the processed net sample, thereby attributing detected activity to Symbiodinium as endosymbionts within planktonic hosts. However, due to the concentrating nature of the net haul, especially in highly productive marine areas such as the northern Adriatic, even cells smaller than the net mesh size may be included in the net sample (Baricevic et al. 2024; Grižančić et al. 2023). The free‐living and symbiotic stages of *Symbiodinium* will need to be confirmed by microscopy observations in future studies to draw reliable conclusions about the origin of metabolic activity (free‐living or endosymbiotic *Symbiodinium*). Currently, our findings suggest that *Symbiodinium* may play a significant role in the overall metabolic activity of the northern Adriatic. However, whether this role is sustained by free‐living cells, endosymbionts, or both remains to be determined. Confirmation of *Symbiodinium* further supports the existence of previously undiscovered, and, due to detected expression activity, likely important contributors to the diversity and functioning of the northern Adriatic. How this high expression relates to nutrient cycling and primary production in the region is yet to be explored at larger sampling scales (perennial), which would provide more reliable insights into the physiological patterns of *Symbiodinium* in the northern Adriatic ecosystem. Additionally, combining phytoplankton and zooplankton data would help identify possible *Symbiodinium* hosts and better define trophic interactions. It is expected that the environmental roles of various *Symbiodinium* life forms will differ, as in hospite *Symbiodinium* provides products of photosynthesis to its host while receiving inorganic nutrients and dissolved organic materials from the host, whereas free‐living *Symbiodinium* in the water column is likely to experience low inorganic nutrient conditions that are unfavourable for photosynthesis, potentially activating a heterotrophic feeding strategy (Jeong et al. 2012).

Notably, even when *Symbiodinium* is excluded from the dinoflagellate community, dinoflagellates remain the most metabolically active phylum among phytoplankton size fraction greater than 50 μm, as shown by the proportion of inter‐phylum TPM normalised values attributed to them in each sample. In metatranscriptomic studies of marine microbial eukaryotes, this pattern has often been observed in oligotrophic ocean regions (Cohen et al. 2021; Lampe et al. 2019). In the northern Adriatic, recent metabarcoding‐based studies have also observed dominance of dinoflagellate ASVs at some stations throughout the year, not only in summer (Armeli Minicante et al. 2019; Grižančić et al. 2023). The same pattern was recently observed in oligotrophic waters of the southern Adriatic (Baricevic et al. 2024). However, it is important to note that dinoflagellates have exceptionally large genomes, and genomics and expressed sequence tag (EST) data suggest that only approximately 10%–27% of dinoflagellate genes are regulated transcriptionally (Lin 2011). This consideration might also influence the number of ORFs detected for dinoflagellates. Whether the observed metabolic dominance of dinoflagellates is due to their unique biology or an increase in their relative abundance within the phytoplankton community remains uncertain. Further research combining multiple species detection methods (e.g., light microscopy and metabarcoding) with a metatranscriptomic approach is needed to clarify this. Additionally, the observed dinoflagellate dominance should be considered in the context of a phytoplankton net sampling bias towards larger (micro‐sized) phytoplankton species, resulting in only a fraction of the eukaryotic plankton community being sampled. Other, possibly critical, members within the nano‐ and pico‐eukaryotic size classes were likely missed or under‐sampled. Accordingly, for now, we can only conclude that the majority of the analysed net phytoplankton community metabolism during the observed period can be attributed to dinoflagellates, while other net‐biased phytoplankton groups may also have contributed notably but were unrecorded here.

Succession patterns of diatoms and coccolithophores align well with previous studies. Diatoms (Bacillariophyta), along with other Ochrophytes, showed elevated relative expression values during autumn, which is consistent with previous studies detecting a characteristic autumn bloom of diatoms (Aubry et al. 2004; Godrijan et al. 2013; Marić et al. 2012; Totti et al. 2019). The absence of a spring diatom and the corresponding dinoflagellate dominance can be considered exceptional compared with previous studies (Godrijan et al. 2013; Marić et al. 2012; Vlašiček et al. 2025). In 2021, the spring bloom was likely suppressed or terminated by a *Noctiluca* bloom. Haptophytes have been reported in the literature as most abundant during winter (December–February) and in May–June (Cerino et al. 2017), which perfectly aligns with our data, describing two Haptophyta activity peaks: first in May on station RV004 and second in February on both stations. Cryptophytes and chlorophytes have not been studied in the northern Adriatic to the same extent as the previously mentioned phyla, and further research is needed to establish annual succession patterns. Therefore, the activity patterns of these phyla detected in our study provide valuable new insights. Both phyla showed high activity during most of the sampling period (except during the spring *Noctiluca* bloom), exhibiting stable expression patterns without pronounced activity peaks. Overall, the annual phytoplankton taxonomic succession detected with metatranscriptomics showed a similar pattern at both sampling stations, indicating that the two stations share characteristics that support phytoplankton (especially dinoflagellate) community structure. These shared characteristics for the two sampling stations (RV001 and RV004) were also confirmed by long‐term monitoring parameters.

### 4.2 Phytoplankton Community Ordination Patterns

A NMDS analysis of the entire phytoplankton community indicated seasonal patterns of functional succession (Figure 12). Spring samples formed the most distinct cluster, with only one sample from station RV004 in April positioned further apart. The observed community functional ordination pattern corresponds to the spring dominance of *Noctiluca*, when the activity of all other phyla was reduced. This is particularly evident in the sample from station RV004 in April, where *Noctiluca* accounted for a significant proportion of annotated ORFs. However, the contribution of other dinoflagellate genera (including *Amphidinium*, *Azadinium*, *Dinophysis*, *Gambierdiscus*, *Gymnodinium*, *Heterocapsa*, *Karenia*, *Lingulodinium*, *Prorocentrum* and *Togula*) to the separation of the spring cluster should not be overlooked, as they also showed higher activity in spring. Winter and autumn samples also showed strong clustering, with the sample from station RV004 in October being distant from other autumn samples. Notably, this sample displayed the highest peak of diatom activity, indicating the presence of an autumn diatom bloom and its specific metabolic fingerprint. Summer samples were the most dispersed, suggesting that overall phytoplankton metabolic activity during this period is most variable. Phylum‐specific NMDS analysis is consistent with the whole phytoplankton community NMDS analysis (Figure 11). Although the April and October RV004 samples stand out from their seasonal clusters and differ from the RV001 samples for the same months, the station parameter had limited influence on sample ordination, indicating very similar expression patterns at stations RV001 and RV004 throughout the year and across all detected phytoplankton phyla.

The RDA models produced different results for the targeted phyla (Figure 11). The highest proportion of variance explained by environmental parameters was observed for Dinoflagellata, whereas for all other phyla, the first two RDA axes together accounted for less than 20% of the variance. This suggests that the included environmental factors had the strongest influence on dinoflagellate functional composition, while other factors likely played a more significant role in shaping the remaining phyla. Additionally, no environmental variables showed strong directional alignment with spring and autumn samples for any of the selected phyla, suggesting that factors influencing phyla composition during these seasons are not captured by the variables selected in this study. These factors could include unmeasured environmental variables (such as meteorological conditions, wind and current regimes, river and rainwater inputs) or biological interactions (such as inter‐species competition and predation). In line with this, spring samples were characterised by the metabolic dominance of a *Noctiluca* bloom. A study on *Noctiluca* blooms in the North Atlantic and the Black Sea reported a significant negative correlation between Noctiluca abundance and wind intensity, suggesting a direct influence of wind on bloom dynamics (Mikaelyan et al. 2014). The authors proposed that *Noctiluca* outbreaks during spring are primarily driven by wind conditions. An unmeasured environmental variable influencing the observed community ordination could be the pressure exerted by dinoflagellate dominance, which has a greater impact on the accompanying phytoplankton community than abiotic environmental parameters. Furthermore, the periodic influence of oligotrophic southern waters on the northern Adriatic ecosystem, due to circulation patterns and changes in the periodicity of environmental factors, could lead to changes in the succession of phytoplankton assemblages (Cozzi et al. 2020; Vlašiček et al. 2025). Even the ‘mucilage phenomenon’, a well‐known ecological disturbance in the northern Adriatic, could serve as an unmeasured environmental variable for the observed phytoplankton functional clustering. However, the significant time lag (around 20 years) since the last recorded mucilage event (Najdek et al. 2002) reduces the likelihood of a connection.

Still, most of the included environmental variables appeared to be significant predictors, except for DIN. This finding aligns with the northern Adriatic being a phosphorus‐limited sea, while nitrogen reserves are generally sufficient for phytoplankton growth (Grilli et al. 2020). This is further supported by Total P being a highly significant predictor of the metabolic activity of all phyla. In the presented RDA models, vectors representing DIN, Total P and Sal are oriented in the same direction, aligning with winter samples. The vector for ${\mathrm{SiO}}_{4}^{-}$ is oriented in the opposite direction, towards late summer samples, and the temperature vector is directed towards summer samples. ${\mathrm{SiO}}_{4}^{-}$ concentration emerged as the most significant predictor for all phyla, not only for Bacillariophyta and other Ochrophyta, as would typically be expected. Since the increase in ${\mathrm{SiO}}_{4}^{-}$ concentration coincides with the autumn diatom bloom, this suggests that competition with Bacillariophyta and other Ochrophytes strongly influenced the broader phytoplankton community structure.

### 4.3 Overview of Dinoflagellate Metabolic Activity Through the Year

#### 4.3.1 Basic Energy Production Pathways

During spring, when *Noctiluca* dominates, there was a clear peak in the expression of metabolic pathways associated with fundamental energy acquisition, including glycolysis, the TCA cycle and pyruvate metabolism, indicating an increased demand for energy production during this period. However, the oxidative phosphorylation pathway showed lower expression in spring. As oxidative phosphorylation requires oxygen, this could suggest oxygen limitation during the *Noctiluca* bloom, potentially due to extensive *Noctiluca* feeding. The elevated expression of the pyruvate metabolism pathway further supports this hypothesis, suggesting a metabolic shift towards anaerobic energy acquisition. Free‐living unicellular algae are among the best‐equipped eukaryotes known for anaerobic energy metabolism (Atteia et al. 2013), and dinoflagellate communities have been found living in various marine anoxic environments (Edgcomb et al. 2002; Stoeck et al. 2009), suggesting that dinoflagellates must possess the metabolic capacity to switch to anaerobic metabolism when oxygen levels are low to support aerobic respiration. This may be particularly useful for species that occur in high biomass and cell density blooms, such as the genus *Noctiluca*.

During this period, increased expression of the Calvin cycle pathway (dark reactions of photosynthesis) was observed, whereas pathways related to light‐dependent photosynthetic reactions (photosynthesis and photosynthesis—antenna proteins) showed lower expression compared with the rest of the year. Notably, the elevated expression of pathways involved in carbohydrate metabolism, such as fructose and mannose metabolism, galactose metabolism and starch and sucrose metabolism, could suggest that the fixed carbon was redirected towards carbohydrate storage and alternative metabolic pathways rather than immediate energy production via light‐dependent photosynthesis. A largely heterotrophic community might explain these findings. A transcriptomic study on processes regulating encystment and dormancy in the dinoflagellate species *Scrippsiella trochoidea* suggests that glycolysis and TCA cycle pathways remained active in resting cysts, while respiration rates were lower and photosynthesis was paused (Deng et al. 2017). A similar metabolic profile (high expression levels of glycolysis and the TCA cycle, with reduced expression of photosynthesis) was observed in our dinoflagellate community during spring, suggesting the presence or formation of dinoflagellate resting stages in the water column.

In summer, after the termination of the *Noctiluca* bloom, pathways associated with photosynthetic light reactions and oxidative phosphorylation show higher expression values, indicating a switch from the prevailing heterotrophic to an autotrophic life strategy, with an increase in primary production and aerobic energy acquisition.

#### 4.3.2 Trophic Strategies

Cellular process pathways related to phagotrophic feeding, including the phagosome, lysosome and peroxisome, were highly expressed during spring, which was expected given that *Noctiluca* is a phagotrophic species (Fonda Umani et al. 2004). The dominance of phagotrophy in spring is further highlighted by the elevated expression of several protease genes, such as cathepsin proteases, cysteine endopeptidase and calreticulin. These proteins and genes have previously been described as important in molecular studies on actively preying protists (Gotthardt et al. 2002; Labarre et al. 2020). However, some phagotrophy‐related pathways remained highly expressed throughout the year, particularly those involved in endocytosis and regulation of the actin cytoskeleton, together with high expression of individual cytoskeletal components, supporting the suggested wide range of survival strategies in northern Adriatic phytoplankton (Ivančić et al. 2012, 2016). Actin, tubulin and myosin‐related genes were among the 60 genes with the highest mean expression values, showing peak expression in winter. In transcriptomic and metatranscriptomic studies on mixotrophic and heterotrophic flagellates under grazing conditions, cytoskeletal components were upregulated and associated with phagocytosis (Labarre et al. 2020; McKie‐Krisberg et al. 2018). In a recent metatranscriptomic study of dinoflagellate communities in the open ocean, elevated expression of cathepsins, cysteine peptidases, calreticulin and actin and tubulin components was found in mesopelagic zones, where photosynthesis genes were downregulated (Cohen et al. 2021).

Pathways associated with light‐dependent photosynthetic reactions showed higher expression in late spring and remained elevated throughout summer and autumn, indicating increased primary production during this period. The significance of photosynthesis as a trophic strategy in dinoflagellates is further supported by the consistently high expression of α‐carbonic anhydrase, recognised as an important component of the carbon‐concentrating mechanism (CCM) in marine eukaryotic phytoplankton (Dimario et al. 2018; Giordano et al. 2005; Jensen et al. 2019; Reinfelder 2011).

Both photosynthesis and phagotrophy emerged as important trophic strategies for dinoflagellates, though they were utilised during different periods of the annual cycle. Phagotrophy was dominant in spring, particularly during the *Noctiluca* bloom, and in winter when daylight duration was shorter and temperatures were lower, highlighting the predominance of secondary production during that period. In contrast, photosynthesis was widely utilised during summer and autumn, the warmer months with greater light availability and consistently high nutrient availability.

#### 4.3.3 Cell Cycle

The observed seasonal variation in Genetic Information Processing pathways indicates distinct shifts in the metabolic priorities of the dinoflagellate community throughout the year. In spring, the upregulation of pathways associated with basal transcription factors, DNA replication, RNA polymerase and RNA degradation suggests increased transcriptional and translational activity, likely supporting rapid cell proliferation and bloom formation. In contrast, during summer, autumn and winter, despite a slight elevation in early autumn, the predominant expression of pathways related to the spliceosome, ribosome, nucleocytoplasmic transport, protein export and protein processing in the endoplasmic reticulum suggests a shift towards post‐transcriptional regulation, protein homeostasis and cellular maintenance. This transition may reflect an adaptive strategy favouring long‐term survival and efficient resource utilisation outside bloom periods. This is further supported by the observation that dinoflagellate metabolic activity in the community decreased after the termination of the *Noctiluca* bloom, but cumulative expression levels remained relatively constant throughout the rest of the year.

Collectively, these findings highlight a seasonal shift in metabolic investment, with spring favouring rapid population expansion and secondary production, while the rest of the year is characterised by cellular maintenance and adaptation, aligning with changes in trophic strategy and environmental conditions.

#### 4.3.4 Environmental Information Processing

The seasonal patterns observed in Environmental Information Processing pathways suggest distinct shifts in the trophic strategy, cellular function and response to environmental stressors within the dinoflagellate community. Spring was marked by four environmental information processing pathways having high transcription levels: ABC transporters, MAPK signalling pathway—yeast, phosphatidylinositol signalling system and mTOR signalling pathways. The possible role of highly expressed ATP‐binding cassette (ABC) transporters in dinoflagellates during spring could be environmentally linked to various processes, as ABC transporters are involved in the active transport of a wide variety of substrates across different types of cellular membranes (Jones and George 2004). Studies on ABC transporters in microorganisms are scarce, and their functions are not well understood. In the coastal bacterioplankton community, ABC transporters were associated with dissolved organic carbon (DOC) transport, facilitating access to the coastal DOC pool as part of the heterotrophic life strategy of bacterioplankton (Poretsky et al. 2010). Several ABC transporter genes have been found in dinoflagellates (Yang et al. 2011) and their roles were primarily related to transport or sequestration of endogenous secondary metabolites and xenobiotic pollutants, but it was also proposed that ABC transporters export polysaccharides outside of dinoflagellate cells (Gong et al. 2017; Gu et al. 2019). During early summer and autumn, the two‐component system, MAPK signalling pathway and MAPK signalling pathway—plant, were highly expressed while during late autumn and winter calcium, cAMP and cGMP‐PKG signalling pathways showed higher expression levels. Transduction of signal from the cell surface into genome using cAMP‐dependent pathway has been confirmed in unicellular eukaryotes and activators of this pathway included stress factors, nutrients or some biologically active substances (Shemarova 2009). Further, in dinoflagellates, the role of cAMP in the regulation of the dinoflagellate cell cycle was confirmed (Lam et al. 2001). Overall, the observed differences in environmental sensing highlight a dynamic seasonal utilisation of basic energy metabolism, trophic strategies and rapid proliferation during bloom periods, in contrast to cell cycle regulation and maintenance, with the potential to detect nutrient pulses typical for the region (Ivančić et al. 2012, 2016).

#### 4.3.5 Nutrient Metabolism

During spring, no nitrate or nitrite transporters showed consistently high expression levels. However, elevated expression compared with the rest of the year was recorded for nitrite reductases (nirB and nirD). This pattern may reflect intracellular nitrogen recycling, metabolic preparedness for shifts in nitrogen availability or potentially the processing of residual intracellular nitrogen originating from earlier nitrate and nitrite uptake. Notably, the continuously elevated expression of Amt family ammonium transporters and urease, which converts urea into ammonium, suggests that ammonium may serve as the primary nitrogen source during this period (Bhovichitra and Swift 1977; Glibert et al. 2016; Thompson et al. 1989). Ammonium easy uptake and incorporation into biomass (compared with nitrate) has favourable energetics for dinoflagellates and enables their high growth rate (Glibert et al. 2016) as it was observed in spring, primarily for *Noctiluca*, but also for some other dinoflagellates. Since oceanographic data confirm nitrate availability during spring along with the ammonium, further indicates preferred ammonium over nitrogen uptake of dinoflagellate community during spring. Moreover, ammonium is known to suppress the expression of nitrate and nitrite transporters in many phytoplankton species, which could also explain the low nitrate and nitrite transporter expression (Cochlan and Harrison 1991; Dortch 1990; Glibert et al. 2016; L'Helguen et al. 2008). This ammonium availability that most likely sustained dinoflagellate high growth rate in the spring, was probably regenerated in situ (from zooplankton excretion or bacterial remineralization in the water column or sediment) (Glibert et al. 2016) or entered as natural or anthropogenic nutrient input (Cozzi et al. 2020) during previous months. An additional advantage for intense dinoflagellate growth in spring likely results from consistently high expression of several genes involved in phosphorus metabolism during this season, compared with the rest of the year. In spring, inorganic phosphate transporters, genes associated with phosphorus acquisition from organic sources (alkaline and acid phosphatases), polyphosphate hydrolysis (exophosphatase), inorganic pyrophosphate hydrolysis (inorganic pyrophosphatase) and membrane lipid remodelling (phospholipase D) were all highly expressed. These pathways have been identified in transcriptomic and metatranscriptomic studies, as well as in broader research on dinoflagellate ecology, highlighting their role as key adaptations for alternative phosphorus metabolism (Lin et al. 2016, 2011; Morey et al. 2011). The activity of a wide spectrum of mechanisms for phosphate acquisition reflects the successful adaptability of dinoflagellates to the almost permanent phosphorus deficiency in the northern Adriatic coastal marine environment (Ivančić et al. 2012, 2016) that was confirmed in this study as well. Similarly, during summer, when lowest nutrient availability was recorded, genes encoding both high and low‐affinity phosphate transporters were highly expressed alongside alkaline phosphatases and phospholipases, indicating that dinoflagellates employed multiple phosphorus acquisition and recycling strategies during this period as well. Interestingly, from late spring through summer, a noticeable drop in surface water salinity was observed, indicating the intrusion of low‐salinity water from the Po River, but without any evident contribution to overall nutrient availability in the studied region during the summer. In late autumn and winter, genes involved in nitrogen assimilation and utilisation exhibited higher expression than during the rest of the year. DIN concentrations peaked in November and December at both sampling stations. The greater availability of nutrients and the most significant increases in the N:P ratio in late autumn and early winter were also confirmed in previous studies for the northern Adriatic (Cozzi et al. 2020; Degobbis et al. 2000) and were explained with mild meteorological conditions and an increase in the precipitation and river runoff in that period of the year. In December, there was an increase in the expression of nitrate reductase, ferredoxin‐nitrate reductase and nitrite reductase (NAD(P)H), followed by elevated expression of genes associated with nitrogen assimilation into amino acids, including glutamine synthetase, glutamate synthase and glutamate dehydrogenase. These findings suggest that dinoflagellates successfully employ strategies for rapid uptake of newly available inorganic nitrogen and its incorporation into cellular components when environmental conditions permit. During winter, DOP concentrations also remained high, and an atypical dual‐specificity phosphatase showed consistently high expression. Similarly, a phosphate transporter from the MFS family exhibited the same expression pattern, suggesting that dinoflagellates actively utilise both organic and inorganic phosphorus sources during the winter period.

## 5 Conclusion

The northern Adriatic is a highly dynamic ecosystem, where multiple environmental stressors, particularly phosphorus limitation, influence phytoplankton growth. While many studies have established annual succession models and examined phytoplankton physiology in response to phosphorus availability, to our knowledge, metatranscriptomic analyses of northern Adriatic phytoplankton have been lacking. This study applied a metatranscriptomic approach to determine annual succession patterns in the taxonomic and functional composition of the phytoplankton community, with a particular focus on dinoflagellates, which atypically predominated during the observation period. Dinoflagellate metabolic dominance was detected throughout the year. Peaks in metabolic activity of well‐known northern Adriatic dinoflagellate genera align with succession patterns established in previous studies based on light microscopy. Additionally, the detection of high metabolic activity assigned to genera whose ecology is unknown in the studied area, such as the genus *Symbiodinium*, suggests a previously overlooked contribution to phytoplankton diversity and ecosystem functioning. Further research, including long‐term datasets of both phyto‐ and zooplankton data along with environmental parameters, is necessary to reveal ecosystem roles and validate their importance in the northern Adriatic, especially given the symbiotic potential of the genus *Symbiodinium*. The underlying causes of dinoflagellate dominance, whether a result of their unique biological traits, stochastic events, or an emerging trend in their relative abundance, remain to be clarified by further research. Community ordination patterns suggested distinct seasonal trends, which also align with previous studies. These findings underscore the importance of environmental factors, such as nutrient availability, in shaping phytoplankton community composition, with notable influences of phosphorus and silicon concentrations throughout the year. However, our results also indicate the importance of unmeasured environmental or biological variables that could have influenced the functional community structure. The distinct seasonal variations in pathway expression observed in dinoflagellates provide valuable insights into their metabolic and cellular adaptations to changing environmental conditions and possibly the underlying mechanisms leading to a positive trend in dinoflagellate relative abundance in the north‐eastern Adriatic. This study revealed seasonal shifts in energy production pathways, trophic strategies, cell cycle and nutrient acquisition and utilisation. During spring, metabolic activity was directed towards energy production through glycolysis and the TCA cycle, while a shift towards oxidative phosphorylation occurred in summer, suggesting a transition from anaerobic to aerobic metabolism. Both photosynthesis and phagotrophy emerged as important trophic strategies for dinoflagellates, with distinct temporal patterns during the annual cycle. Phagotrophy dominated in spring, particularly during the *Noctiluca* bloom, and in winter when daylight duration was shorter and temperatures were lower, while photosynthesis became more prominent in the warmer months with greater light availability. Nitrogen and phosphorus metabolism also exhibited clear seasonal trends, with dinoflagellates employing various strategies for nutrient acquisition and recycling, depending on the availability of these resources. Changes observed in the activation of different cellular processes highlight a seasonal shift in metabolic investment, with spring favouring rapid population expansion, while the rest of the year is characterised by cellular maintenance and adaptation. Overall, our findings highlight the intricate interplay between environmental factors, metabolic strategies and seasonal succession in the northern Adriatic phytoplankton community. This study provides critical new insights into dinoflagellate ecology, but also emphasises the need for further multi‐method research to fully understand their role in the northern Adriatic ecosystem.

## Author Contributions

**Mia Knjaz:** conceptualization (equal), formal analysis (equal), investigation (equal), methodology (equal), visualization (equal), writing – original draft (lead), writing – review and editing (supporting). **Ana Baricevic:** conceptualization (equal), formal analysis (equal), investigation (equal), methodology (equal), supervision (equal), writing – original draft (supporting), writing – review and editing (lead). **Mirta Smodlaka Tankovic:** conceptualization (equal), data curation (equal), funding acquisition (equal), investigation (equal), resources (equal), writing – original draft (supporting). **Natasa Kuzat:** data curation (supporting), investigation (supporting), methodology (supporting). **Ivan Vlasicek:** formal analysis (supporting), investigation (equal), methodology (supporting), visualization (supporting). **Lana Grizancic:** formal analysis (supporting), investigation (supporting), methodology (equal), visualization (supporting). **Ivan Podolsak:** data curation (supporting), formal analysis (supporting), investigation (supporting). **Tjasa Kogovsek:** conceptualization (equal), formal analysis (supporting), investigation (equal). **Ariana Turkovic:** data curation (supporting), formal analysis (supporting), investigation (supporting). **Martin Pfannkuchen:** conceptualization (equal), formal analysis (equal), funding acquisition (equal), investigation (equal), methodology (equal), visualization (equal), writing – original draft (supporting). **Daniela Maric Pfannkuchen:** conceptualization (equal), funding acquisition (lead), investigation (equal), project administration (lead), supervision (equal), writing – original draft (supporting), writing – review and editing (supporting).

## Funding

This work was supported by H2020 Environment, JERICO‐S3 and Hrvatska Zaklada za Znanost, UIP‐2014‐09‐6563, UIP‐2020‐02‐7868.

## Disclosure

Benefits from this research accrue from the sharing of our data and results on public databases as described above.

## Conflicts of Interest

The authors declare no conflicts of interest.

## Supporting information


**Table S1:** ece372835‐sup‐0001‐TablesS1‐S4.docx.

## Data Availability

Raw sequence reads and sample metadata are deposited in the European Nucleotide Archive (ENA) at EMBL‐EBI under accession number PRJEB87874.
