# Supplementary material for: Revealing the Physiological Patterns of Dinoflagellates in North‐Eastern Adriatic Phytoplankton
Source: Ecol Evol. 2026 Jan 4;16(1):e72835. doi: 10.1002/ece3.72835 (PMC12765665; doi:10.1002/ece3.72835)
Supplement: Supplementary file 1 — Table S1: ece372835‐sup‐0001‐TablesS1‐S4.docx. [file ECE3-16-e72835-s001.docx]

## Tables and figures

### Supplementary tables

Supplementary table 1 **Results of the PERMANOVA analysis examining the effect of season on phytoplankton community composition at the phylum level.** The table includes degrees of freedom (Df), sum of squares (SumsOfSqs), mean squares (MeanSqs), F-statistic (F.Model), proportion of variance explained (R²), and significance (Pr(>F)). Significant results (p ≤ 0.05) indicate a strong seasonal influence on phytoplankton composition.

|  | **PERMANOVA RESULTS - season** | | | | | | |
| --- | --- | --- | --- | --- | --- | --- | --- |
| **Phylum** | **Factor** | **Df** | **SumsOfSqs** | **MeanSqs** | **F.Model** | **R^2^** | **Pr(>F)** |
| **Phytoplankton (6 Phyla)** | season | 3 | 0.464620627 | 0.154874 | 5.052525 | 0.431133 | 0.001 |
|  | Residuals | 20 | 0.613054064 | 0.030653 | NA | 0.568867 | NA |
|  | Total | 23 | 1.077674691 | NA | NA | 1 | NA |
| **Dinoflagellata** | season | 3 | 1.228054712 | 0.409352 | 18.13328 | 0.731182 | 0.001 |
|  | Residuals | 20 | 0.451492093 | 0.022575 | NA | 0.268818 | NA |
|  | Total | 23 | 1.679546804 | NA | NA | 1 | NA |
| **Bacillariophyta** | season | 3 | 1.978994012 | 0.659665 | 5.919134 | 0.470303 | 0.001 |
|  | Residuals | 20 | 2.22892288 | 0.111446 | NA | 0.529697 | NA |
|  | Total | 23 | 4.207916892 | NA | NA | 1 | NA |
| **Other_Ochrophyta** | season | 3 | 1.192027064 | 0.397342 | 3.469818 | 0.34231 | 0.001 |
|  | Residuals | 20 | 2.290277631 | 0.114514 | NA | 0.65769 | NA |
|  | Total | 23 | 3.482304695 | NA | NA | 1 | NA |
| **Haptophyta** | season | 3 | 1.08287915 | 0.36096 | 3.403014 | 0.337947 | 0.001 |
|  | Residuals | 20 | 2.121411958 | 0.106071 | NA | 0.662053 | NA |
|  | Total | 23 | 3.204291108 | NA | NA | 1 | NA |
| **Chlorophyta** | season | 3 | 0.727682207 | 0.242561 | 3.818087 | 0.364156 | 0.001 |
|  | Residuals | 20 | 1.270587875 | 0.063529 | NA | 0.635844 | NA |
|  | Total | 23 | 1.998270082 | NA | NA | 1 | NA |
| **Cryptophyta** | season | 3 | 1.029521993 | 0.343174 | 3.325954 | 0.332841 | 0.001 |
|  | Residuals | 20 | 2.06361247 | 0.103181 | NA | 0.667159 | NA |
|  | Total | 23 | 3.093134463 | NA | NA | 1 | NA |

Supplementary table 2 **Results of the PERMANOVA analysis assessing the effect of station on phytoplankton community composition at the phylum level.** The table includes degrees of freedom (Df), sum of squares (SumsOfSqs), mean squares (MeanSqs), F-statistic (F.Model), proportion of variance explained (R²), and significance (Pr(>F)). The analysis indicates that station has no significant effect on phytoplankton composition, as all p-values are non-significant (Pr(>F) > 0.05).

|  | **PERMANOVA RESULTS - station** | | | | | | |
| --- | --- | --- | --- | --- | --- | --- | --- |
| **Phylum** | **Factor** | **Df** | **SumsOfSqs** | **MeanSqs** | **F.Model** | **R^2^** | **Pr(>F)** |
| **Phytoplankton (6 Phyla)** | station | 1 | 0.018948 | 0.018948 | 0.393743 | 0.017583 | 0.999 |
|  | Residuals | 22 | 1.058726 | 0.048124 | NA | 0.982417 | NA |
|  | Total | 23 | 1.077675 | NA | NA | 1 | NA |
| **Dinoflagellata** | station | 1 | 0.011322 | 0.011322 | 0.149315 | 0.006741 | 0.995 |
|  | Residuals | 22 | 1.668224 | 0.075828 | NA | 0.993259 | NA |
|  | Total | 23 | 1.679547 | NA | NA | 1 | NA |
| **Bacillariophyta** | station | 1 | 0.063691 | 0.063691 | 0.338111 | 0.015136 | 0.999 |
|  | Residuals | 22 | 4.144226 | 0.188374 | NA | 0.984864 | NA |
|  | Total | 23 | 4.207917 | NA | NA | 1 | NA |
| **Other_Ochrophyta** | station | 1 | 0.078326 | 0.078326 | 0.506221 | 0.022493 | 0.991 |
|  | Residuals | 22 | 3.403979 | 0.154726 | NA | 0.977507 | NA |
|  | Total | 23 | 3.482305 | NA | NA | 1 | NA |
| **Haptophyta** | station | 1 | 0.066721 | 0.066721 | 0.467837 | 0.020823 | 0.999 |
|  | Residuals | 22 | 3.13757 | 0.142617 | NA | 0.979177 | NA |
|  | Total | 23 | 3.204291 | NA | NA | 1 | NA |
| **Chlorophyta** | station | 1 | 0.03584 | 0.03584 | 0.401784 | 0.017935 | 0.996 |
|  | Residuals | 22 | 1.96243 | 0.089201 | NA | 0.982065 | NA |
|  | Total | 23 | 1.99827 | NA | NA | 1 | NA |
| **Cryptophyta** | station | 1 | 0.087387 | 0.087387 | 0.639613 | 0.028252 | 0.938 |
|  | Residuals | 22 | 3.005747 | 0.136625 | NA | 0.971748 | NA |
|  | Total | 23 | 3.093134 | NA | NA | 1 | NA |

Supplementary table 3 **Results of the Redundancy Analysis (RDA) model significance test for phytoplankton community composition at the phylum level, assessed using ANOVA.** The table includes degrees of freedom (Df), variance explained, F-value, and significance (Pr(>F)). Significance codes: 0 ‘***’, 0.001 ‘**’, 0.01 ‘*’, 0.05 ‘.’ and 0.1 ‘ ’.

| **Phylum** | **Factor** | **Df** | **Variance** | **F-value** | **Pr(>F)** | **Significance** |
| --- | --- | --- | --- | --- | --- | --- |
| **Dinoflagellata** | Model | 5 | 13245.4 | 4.8216 | 0.001 | *** |
|  | Residual | 18 | 9889.6 |  |  |  |
| **Bacillariophyta** | Model | 5 | 6656.6 | 1.4731 | 0.001 | *** |
|  | Residual | 18 | 16267.2 |  |  |  |
| **Other_Ochrophyta** | Model | 5 | 8784.5 | 1.5549 | 0.001 | *** |
|  | Residual | 18 | 20339 |  |  |  |
| **Haptophyta** | Model | 5 | 8153.4 | 1.6116 | 0.001 | *** |
|  | Residual | 18 | 18213.1 |  |  |  |
| **Chlorophyta** | Model | 5 | 8675.7 | 1.6808 | 0.001 | *** |
|  | Residual | 18 | 18581.5 |  |  |  |
| **Cryptophyta** | Model | 5 | 8664.4 | 1.573 | 0.001 | *** |
|  | Residual | 18 | 19829 |  |  |  |

Supplementary table 4 **Results of the Redundancy Analysis (RDA) model significance test for phytoplankton community composition at the phylum level, assessing the influence of different environmental variables, using ANOVA.** Environmental variables included temperature (°C), dissolved inorganic nitrogen concentration (DIN), orthosilicate concentration (SiO_4_), salinity (Sal), and total phosphorus concentration (Total_P). The table includes degrees of freedom (Df), variance explained, F-value, and significance (Pr(>F)). Significance codes: 0 ‘***’, 0.001 ‘**’, 0.01 ‘*’, 0.05 ‘.’ and 0.1 ‘ ’.

| **Phylum** | **Environmental Variable** | **Df** | **Variance** | **F-value** | **Pr(>F)** | **Significance** |
| --- | --- | --- | --- | --- | --- | --- |
| **Dinoflagellata** | DIN | 1 | 572.6 | 1.0422 | 0.327 |  |
|  | SiO₄ | 1 | 5733.5 | 10.4355 | 0.001 | *** |
|  | Total_P | 1 | 1811.9 | 3.2977 | 0.028 | * |
|  | °C | 1 | 3879.8 | 7.0615 | 0.002 | ** |
|  | Sal | 1 | 1247.6 | 2.2708 | 0.07 | . |
|  | Residual | 18 | 9889.6 |  |  |  |
| **Bacillariophyta** | DIN | 1 | 1074.8 | 1.1893 | 0.12 |  |
|  | SiO₄ | 1 | 1504.1 | 1.6643 | 0.001 | *** |
|  | Total_P | 1 | 1434.5 | 1.5873 | 0.002 | ** |
|  | °C | 1 | 1299.2 | 1.4376 | 0.007 | ** |
|  | Sal | 1 | 1343.9 | 1.4871 | 0.005 | ** |
|  | Residual | 18 | 16267.2 |  |  |  |
| **Other_Ochrophyta** | DIN | 1 | 1308.8 | 1.1583 | 0.13 |  |
|  | SiO₄ | 1 | 2137.6 | 1.8918 | 0.001 | *** |
|  | Total_P | 1 | 1815.3 | 1.6066 | 0.001 | *** |
|  | °C | 1 | 1670.2 | 1.4781 | 0.006 | ** |
|  | Sal | 1 | 1852.6 | 1.6395 | 0.002 | ** |
|  | Residual | 18 | 20339 |  |  |  |
| **Haptophyta** | DIN | 1 | 1162.8 | 1.1492 | 0.139 |  |
|  | SiO₄ | 1 | 2156.7 | 2.1314 | 0.001 | *** |
|  | Total_P | 1 | 1661 | 1.6415 | 0.001 | *** |
|  | °C | 1 | 1580.3 | 1.5618 | 0.005 | ** |
|  | Sal | 1 | 1592.7 | 1.574 | 0.001 | *** |
|  | Residual | 18 | 18213.1 |  |  |  |
| **Chlorophyta** | DIN | 1 | 1205 | 1.1673 | 0.167 |  |
|  | SiO₄ | 1 | 2222.4 | 2.1529 | 0.001 | *** |
|  | Total_P | 1 | 1784.4 | 1.7285 | 0.004 | ** |
|  | °C | 1 | 1657.4 | 1.6056 | 0.004 | ** |
|  | Sal | 1 | 1806.5 | 1.75 | 0.001 | *** |
|  | Residual | 18 | 18581.5 |  |  |  |
| **Cryptophyta** | DIN | 1 | 1241.1 | 1.1266 | 0.188 |  |
|  | SiO₄ | 1 | 2203.2 | 1.9999 | 0.001 | *** |
|  | Total_P | 1 | 1771.5 | 1.6081 | 0.003 | ** |
|  | °C | 1 | 1643.3 | 1.4917 | 0.006 | ** |
|  | Sal | 1 | 1805.4 | 1.6389 | 0.003 | ** |
|  | Residual | 18 | 19829 |  |  |  |
